# Evidence in disease and non-disease contexts that nonsense mutations cause altered splicing via motif disruption

**DOI:** 10.1093/nar/gkab750

**Published:** 2021-09-01

**Authors:** Liam Abrahams, Rosina Savisaar, Christine Mordstein, Bethan Young, Grzegorz Kudla, Laurence D Hurst

**Affiliations:** The Milner Centre for Evolution, Department of Biology and Biochemistry, University of Bath, Bath BA2 7AY, UK; The Milner Centre for Evolution, Department of Biology and Biochemistry, University of Bath, Bath BA2 7AY, UK; Instituto de Medicina Molecular João Lobo Antunes, Faculdade de Medicina, Universidade de Lisboa, 1649-028 Lisboa, Portugal; The Milner Centre for Evolution, Department of Biology and Biochemistry, University of Bath, Bath BA2 7AY, UK; MRC Human Genetics Unit, The University of Edinburgh, Crewe Road, Edinburgh EH4 2XU, UK; Aarhus University, Department of Molecular Biology and Genetics, C F Møllers Allé 3, 8000 Aarhus, Denmark; MRC Human Genetics Unit, The University of Edinburgh, Crewe Road, Edinburgh EH4 2XU, UK; MRC Human Genetics Unit, The University of Edinburgh, Crewe Road, Edinburgh EH4 2XU, UK; The Milner Centre for Evolution, Department of Biology and Biochemistry, University of Bath, Bath BA2 7AY, UK

## Abstract

Transcripts containing premature termination codons (PTCs) can be subject to nonsense-associated alternative splicing (NAS). Two models have been evoked to explain this, scanning and splice motif disruption. The latter postulates that exonic cis motifs, such as exonic splice enhancers (ESEs), are disrupted by nonsense mutations. We employ genome-wide transcriptomic and *k*-mer enrichment methods to scrutinize this model. First, we show that ESEs are prone to disruptive nonsense mutations owing to their purine richness and paucity of TGA, TAA and TAG. The motif model correctly predicts that NAS rates should be low (we estimate 5–30%) and approximately in line with estimates for the rate at which random point mutations disrupt splicing (8–20%). Further, we find that, as expected, NAS-associated PTCs are predictable from nucleotide-based machine learning approaches to predict splice disruption and, at least for pathogenic variants, are enriched in ESEs. Finally, we find that both in and out of frame mutations to TAA, TGA or TAG are associated with exon skipping. While a higher relative frequency of such skip-inducing mutations in-frame than out of frame lends some credence to the scanning model, these results reinforce the importance of considering splice motif modulation to understand the etiology of PTC-associated disease.

## INTRODUCTION

Understanding the molecular mechanisms that underpin genetic diseases is core to genetic-based medicine (e.g. see (1–3)). Nonsense mutations, generating in-frame premature termination codons (PTCs), are disproportionately common as a cause of genetic disease accounting for around 11.5% of human inherited diseases (4,5). PTC pathogenicity is often assumed to be owing to one of two well-described mechanisms. First, a PTC may result in the synthesis of a truncated protein with potentially problematic loss of function or gain of toxicity (5–7). Second, nonsense-mediated decay (NMD) (8,9) targets some PTC-containing transcripts for degradation, potentially avoiding any toxic effects of truncated proteins in heterozygotes, but largely abolishing expression in PTC homozygotes.

There is, however, at least one further possibility (10), referred to as nonsense-associated altered splicing (NAS) (11–13). Just as synonymous and nonsynonymous mutations can cause disease by altering splicing (14,15), so too PTC-containing exons can cause exon skipping (reviewed in 15). With the PTC bearing exon removed, the PTC in question is hence not subject to NMD (N.B. as PTCs are usually defined at the DNA level under an assumption of canonical splicing, we retain the language of PTCs even if they induce exon skipping).

Skipping of PTC-containing exons can reduce the impact of the PTC if the skipped exon is a multiple of three long (see e.g. 16,17–19) but can also be associated with pathogenicity (see e.g. 20,21–24). Removal of an exon may itself be catastrophic enough to cause disease, even if the exon is a multiple of three long. If the exon skipped is not a multiple of three long, skipping as a result of NAS would introduce a frameshift with the usual deleterious knock-on consequences (novel peptide, downstream PTCs or read-through to the poly A tail).

The mechanism of NAS is unresolved with at least two non-mutually exclusive models being proposed, scanning and motif disruption (for review, see 11,15). The scanning model (25,26) evokes a mechanism that somehow verifies the integrity of an ORF and, when necessary, directs the splicing machinery to skip (or otherwise disrupt the splicing of) the offending exon (reveiwed in 15). Exactly how a nonsense mutation leads to splice disruption in this model is not so clear. One version of the model (25) proposes there to be a machinery for the detection of the PTC via a translation-like scanning mechanism in the nucleus (for review, see 15), potentially comparable with nuclear scanning associated with NMD (27). Although contentious, evidence suggests that both a translation-like mechanism (28–31) and NMD (12) are observed in the nucleus, implying the presence of active ribosomes that could detect PTCs prior to nuclear export. Evidence for coupled transcription and translation in mammalian nuclei (31) adds credence to the possibility of a mechanism permitting translational feedback to co-transcriptional splicing. Other models suggest cytoplasmic scanning via conventional ribosomes with some mode of feedback to splicing (for discussion see 32). Here, PTC recognition occurs during the process of cytoplasmic translation that then acts in trans to increase production of the PTC-free alternatively spliced mRNA. However, how such feedback might occur in a manner that is allele-specific and isoform-specific is far from clear (32).

No matter what the possible mechanism, a defined reading frame is a prerequisite of scanning models (33). Consistent with this, all three nonsense mutations in exon 51 of the FBN1 gene disrupt splicing but regular splicing is restored by introducing frameshifts upstream of the nonsense variant (26) (see also 34,35–37).

Frame dependency has, however, been questioned in some claimed incidences (38), a motif disruption model being argued to be more parsimonious (39). This model suggests that NAS-causing nonsense mutations modulate important splicing regulatory motifs within immature mRNA (11,12). *A priori* nonsense mutations might be more likely than many to disrupt CDS exonic splicing motifs, as such motifs are likely to have an especially low density of stop codons (40) owing to the fact that they are embedded within coding sequence (CDS) (40), which by definition cannot have TGA, TAA or TAG in at least one frame. Indeed, exonic motifs associated with RNA binding proteins typically have a low density of stop codons (40).

In this context, exonic splice enhancers (ESEs) are strong candidates for motifs that might be disrupted by nonsense mutations. ESEs are purine rich motifs that function by binding serine-arginine rich (SR) proteins that in turn direct the splicing machinery to the splice junction and facilitate the assembly of the spliceosome (41). Mutational disruption of ESEs resulting in incorrect splicing is well described (11,22,42–47) and ESEs are especially abundant at exon ends (48), the terminal ≈ 70bp (48), where splice disrupting mutations are most common (49). ESEs may also exist in a sequence space that is especially prone to disruptive nonsense mutations. Despite the purine enrichment of both ESEs and stop codons, ESEs have a low density of TAA, TGA and TAG motifs (40) (see also 50). This may well reflect the fact that, while such motifs need only avoid nonsense mutations in one frame, they tend to be employed in all frames (51). ESEs then may well have a high rate of gain of nonsense mutations owing to purine richness, while the same nonsense mutations are likely to destroy the ESE, owing to ESE being in CDS and hence having a low TGA, TAA or TAG density.

The motif disruption model presumes that a point mutation that disrupts the motif can be of large enough effect to make meaningful differences to splice patterns. This is supported by population genetic and molecular evolutionary analyses (48,52–57), by individual case histories (see for review 15,41) and by minigene random mutagenesis experiments (for meta-analysis see 58). Such point mutations are known to be causative of genetic disease (see, e.g. 20,59,60–63). Recent population genetic evidence also indicates that selection on ESE disrupting mutations is commonly strong selection (56). PTCs disrupting ESEs causing NAS have been described (see, e.g. 11,42,43,45,64). Evidence for selection against TAA, TAG and TGA in non-coding transcripts (lncRNAs) owing to selection for ESEs (40), provides further evidence that mutation to these trinucleotides disrupts splicing owing to motif disruption independent of translation (presuming that lncRNAs are not affected by translationally-mediated mechanisms).

While to date NAS has been analysed via close scrutiny of individual examples (for review see 15), here we aim to add to this literature a genome-wide survey. In particular, we scrutinize the motif model as it makes predictions that are approachable by such an approach. We consider three such predictions. First, the model predicts that only some PTCs would cause skipping and, in turn, that rates at which PTCs disturb splicing should be on a par (or slightly higher) than seen for random (non PTC) mutations. Meta-analysis of random mutagenesis experiments involving minigene constructs suggest that on average, allowing for the biased small size of the experimental minigene exons, 8–21% of exonic point mutations disrupt splicing (58). Hence, if nonsense mutations are like any mutations that modulate motifs, then we might expect a similar proportion to also affect splicing. Given that stop codons are depleted in exonic motifs (40), including ESEs (40), the frequency of PTCs that modulate splicing might be expected to be at the upper end of the range seen for random mutations. However, the various estimates for the frequency of splice-disrupting mutations (including ours) are not meaningful at that level of resolution, so we do not broach this issue. We do however, ask whether nonsense mutations are more likely to be associated with skipping than comparable mutations. Second, the motif disruption model predicts which PTCs should (and should not) disrupt splicing. Specifically, the motif model predicts that the PTCs that disrupt splicing are disproportionately embedded in exonic motifs that affect splicing. Third, the motif disruption model predicts that mutations generating the trinucleotides TAA, TGA or TAG in any frame should disrupt splicing as the motifs themselves are frame independent (51).

To address the first prediction, we provide rough estimates for the commonality of PTCs disrupting splicing. We do this by two means, to generate lower and upper bound estimates. To generate a lower bound, we consider the non-disease-associated context via 1000 Genomes data (65) coupled with associated transcriptomics to detect exon skipping associated with PTCs. Note that here we focus on exon skipping alone both because it is the splice disruption mode most reliably detected from the available transcriptomics and because this is the most common mode of splice disruption in wild type state in humans (66), in response to mutation (67) and associated with CRISPR generated indels (68,69). We use a minigene construct to experimentally validate our top NAS candidate (but not to arbitrate on the mechanism). As purifying selection would most likely have removed highly deleterious alleles from the sampled populations prior to analysis, any PTC present in this data is likely not to have major effects. Hence these data most likely under-estimate rates at which *de novo* PTCs are associated with splice disruption. Indeed, a reduced frequency of SNPs at functional ESEs is central to the logic of frequency-based motif confirmation analyses (48). The potential deleterious effects of the PTCs that are observed in such data may be buffered by some means, possibly owing to heterozygosity.

To consider the upper bound, we consider PTCs in the disease-associated context via ClinVar data (70). We expect the frequency of nonsense mutations resulting in NAS to be higher in disease-associated contexts than in non-disease-associated contexts owing to the opposite ascertainment biases. Here we employ an estimation methodology based on enrichment of residues towards exon ends (62,71), splice-disrupting mutations being enriched at ends (48,49). This enrichment analysis is a special case of exon *k*-mer enrichment analysis that is an experimentally validated means to identify splice associated motifs (e.g. 62).

To test the second prediction, we ask whether machine learning models that successfully predict splicing from nucleotide content alone (72) correctly predict which PTCs disrupt splicing the most, as estimated from 1000 Genomes data. As these approaches are ‘blind’ to the underlying mechanism, we also consider specifically whether PTCs are enriched in well-described exonic splicing motifs. For this, we employ ESEs as these are the best-defined exonic splice motifs, with four large scale analyses enabling definition of hexamers that all, or nearly all, analyses agree to be ESEs (52). To examine the third prediction, we employ the same resources as we employed to determine the lower bound estimate (i.e. 1000 genome data with coupled transcriptomics). We estimate the rate at which out-of-frame mutations to TAA, TGA or TAG are also associated with exon skipping. We start by showing that ESEs do indeed sit in an unusual place in sequence space that renders them especially likely to have a high rate of nonsense mutations that in turn break ESE functionality.

## MATERIALS AND METHODS

### Data sources

All analyses were performed using the reference genome sequence and annotations for GRCh37, Ensembl release 87 (73) (http://ftp.ensembl.org/; last accessed 25 January 2018). Polymorphism data was retrieved from the EBI 1000Genomes FTP site (65) (ftp://ftp.1000genomes.ebi.ac.uk/vol1/ftp/, last accessed 24 January 2018). BAM files containing GEM-aligned RNA-seq data for individuals from the 1000 Genomes project were retrieved from the EBI FTP site (74) (http://ftp.ebi.ac.uk/, last accessed 8 February 2018). Only samples present in both datasets were retained. Bam files for the second RNA-seq dataset were downloaded from Array Express (https://www.ebi.ac.uk/arrayexpress/experiments/E-GEOD-19480/, last accessed 25 March 2020). Protein family data was downloaded from Ensembl Biomart (75) (http://grch37.ensembl.org/biomart, last accessed 12 February 2018). ClinVar data containing information regarding disease associated mutations was downloaded from the NCBI FTP site (70) (ftp://ftp.ncbi.nlm.nih.gov/pub/clinvar/, last accessed 11 May 2018). INT3 ESE motifs were retrieved from the supplementary data to Caceres and Hurst (52).

### General methods

Custom Python 3.6.4 scripts were used for all data handling and are available at http://github.com/rosinaSav/NAS_code, including the use of standard Python modules, as well as NumPy v1.91 (76). Data plotting and statistical analyses were performed using R v3.2.1 (77). BEDTools v2.27.1 was used for operations on genome coordinates (78). SAMTools v1.7 was used for BAM file manipulation (79). VCFtools v0.1.15 (80) and tabix v0.2.5 (81) were used to perform operations on SNP data. STAR aligner v2.7 was used to align raw reads from the E-GEOD-19480 dataset (82).

### Analysis of the frequency of nonsense mutations in ESEs

We consider all possible mutations at all possible sites within the INT3 set of hexamers (52) asking whether the mutation would generate an in-frame stop codon where there was none before and if the resulting hexamer is not in turn identified as an ESE within the INT3 list. Specifically, if any given mutation generated a stop codon (in any frame) then this was considered a candidate nonsense mutation. However, in some instances the location of the new stop was an old stop in the original hexamer. For example, TGAAGA is one of the 84 INT3 motifs and a G→A mutation at position 2 generates a new TAA codon (TGAAGA→TAAAGA). In this case, as the original TGA could not have been in-frame this could not be a nonsense mutation and so was excluded. Similarly, if the first trinucleotide is a stop codon then the following trinucleotide cannot also be an in frame nonsense mutation and so was ignored (ie TGAAGA→TGATGA were not considered). Comparably, if the second full codon (residues 4–6) is a stop codon then the first three cannot mutate to an in-frame stop and so these too were not considered. We thus preserved all changes that were from a coding nucleotide, were the frame appropriate, to a stop codon. Significance was determined by repeated sampling of 84 randomly chosen hexamers from concatenation of the full human RefSeq CDS dataset. Each simulant was analysed using the same rules and the number of nonsense mutations determined. *P* was given and *n/m*, where *n* is the number of simulants with as many or more nonsense mutations as in the real data and *m* is the number of simulant data sets (*m *= 10 000).

### Compilation of protein-coding exon set

The main open reading frame (ORF) for protein-coding genes was extracted from the genome annotations. Sequences were filtered to include only those that had canonical start and stop codons, only contained canonical nucleotides, were of a length that is a multiple of three and did not include premature stop codons. Only the transcript isoform with the longest ORF was retained for each of the genes. In order to preserve data independence, only a single gene was retained from each Ensembl protein family, one being selected at random. Finally, the internal fully coding exons that did not overlap other annotated exons were extracted. This filtered set of exons was used for all analyses.

### SNP filtering

SNPs for individuals were intersected with the set of coding exons to obtain all SNPs within the samples. From these, their relative positions within the exon and CDS were calculated. The mutation status of each SNP (e.g. ‘nonsense’ or ‘missense’) was determined with custom Python code using this positional data, and the reference and variant alleles. While focal analysis considers only SNPs that generated PTCs, we also consider out of frame mutations to TGA, TAG and TGA as well as ‘matching’ non-nonsense mutations to compare with the nonsense ones. Note that in the rare event of multiple PTCs being identified in any given exon, to avoid pseudo-replication of data, the exon was considered only once and one PTC selected at random for contextual analysis. This left 1180 PTCs.

### Quantification of splice isoforms

GEM-mapped reads from the Geuvadis BAM files were subject to quality filtering as per the protocol in (74). Briefly, reads were filtered to uniquely mapped reads with a base mapping quality scale between 251 and 255 or 175 and 181 inclusive. Further, only reads with no more than six mismatches were included. These reads were then mapped to the exon-exon junctions that flank the exons in our dataset.

For each exon and each individual, we counted the number of reads that supported inclusion by counting those that mapped to the exon-exon junction between the focal exon and either of the two flanking exons as defined by Ensembl annotations. Similarly, we counted reads supporting skipping by counting the number of reads that mapped to the junction between the two exons flanking the focal exon. The number of reads supporting exon skipping was multiplied by two, as these reads can only map to a single exon-exon junction, whereas reads that support exon inclusion can overlap either of two exon-exon junctions.

Read counts were then used to calculate several metrics for each exon in each sample: PSI, RPMinclude and RPMskip. PSI is defined as the number of reads containing the exon, divided by the number of reads containing the exon plus the number of reads where the exon is skipped. ΔPSI (PSI_PTC−/+_ − PSI_PTC−/−_) is used to describe the PSI difference between the two genotypes for each exon. If there is less exon inclusion when a PTC is present (i.e. lower PSI and increased exon skipping), ΔPSI is negative. We are aware that this custom method for estimating PSI is imperfect, as it may lead to incorrect inferences in the case of splicing aberrations other than exon skipping. However, we could not use existing packages for calculating PSI as our analysis required a metric that could be easily modified to account for the confound of NMD (see below). Exon skipping accounts for the majority of alternative splicing events in humans (66). Therefore, the noise introduced by the imperfections in the method is expected to be small.

RPMinclude is defined as the number of reads containing the exon divided by the total number of reads in the sample. RPMskip is defined as the number of reads without the exon divided by the total number of reads in the sample. Accordingly, ΔRPMincl (RPMinclude_PTC−/+_ − RPMinclude_PTC−/−_) and ΔRPMskip (RPMskip_PTC−/+_ − RPMskip_PTC−/−_) then describe the differences between PTC−/+ and PTC−/− variants for RPMinclude and RPMskip, respectively.

For RPMinclude and RPMskip, the total number of reads remaining after quality filtering of the BAM files is included in the calculation to account for differences in both sequencing depth and read quality between samples. We therefore first determined the total read count. We then filtered the BAM file to only contain reads overlapping our exon-exon junctions. We performed the quality filtering on these exon-exon junction reads and sampled the read count. The proportional decrease between the non-quality filtered exon-exon junction reads and quality filtered exon-exon junction reads was then used to scale the initial read count to estimate the number of total reads after quality filtering. We find no significant difference (*P* = 0.188, paired Wilcoxon signed-rank test) between the proportion of reads retained after filtering the full BAM file and after filtering after intersection with exon−exon junctions, arguing that applying the proportional decrease for exon-exon junctions to the full read count is unbiased and appropriate (see Supplementary Figure S1).

### Further filtering of candidates

1,180 exons were found to contain a PTC in some but not all individuals, allowing for comparison between the different genotypes. Before calculating the metrics described above, we excluded those exons for which less than half of the individuals (with or without the PTC) presented reads mapping to the relevant exon-exon junctions. This was so as to avoid drawing unreliable conclusions based on data from only a small number of individuals.

We also required at least one of the remaining individuals to contain a PTC in the exon (otherwise skipping could not be evaluated) but did no further filtering based on the number of exons within each genotype (PTC+/+, PTC−/− or PTC+/−). This is because by imposing a higher threshold for the minimum number of PTC-containing individuals, we would have biased our selection against the more deleterious PTCs, which are expected to be rare. However, our removal of exons where only a minority of the individuals had reads is expected to have had the side effect of excluding lowly expressed genes. Hence, inferences drawn based on the remaining set are expected to be less sensitive to the number of individuals that they are based on.

In addition, we retained only constitutive exons, defined as exons present in all annotated transcript isoforms. The advantages of this approach are two-fold. First by avoiding noise associated with variable exon skipping in PTC−/− condition the confidence that can be ascribed to any given calls of exon skipping increases. Second, even if skipping rates were perfectly deterministic (not noisy), as native skipping rates go up (i.e. PSI tends to zero), the parameter space within which further skipping can be resolved becomes ever more restricted (at a hypothetical limit of PSI = 0, there can be no further reduction). Thus consideration of exons that appear constitutive renders resolution of changes to rates of skipping, but not of inclusion, maximally robust. This left *N* = 541 PTC-containing exons (for metadata on these exons see Supplementary data S1, for sequences see Supplementary data S2).

### Missense mutation simulations

We performed 100 simulations in which each of the real PTCs was randomly matched to a missense mutation. For each PTC, the missense mutation was sampled in order to match the PTC’s ancestral allele identity, variant allele identity and variant allele frequency (to a precision of 0.05). The same analyses were then performed on the sets of pseudoPTCs (pPTCs). To further control for distance to exon boundary, we calculated the relative PTC position as the distance to the 5′ exon end. We then defined a window of five nucleotides to either side of this position and selected pseudoPTCs whose relative position within the exon in which they were found was within this window. If none were available, the window was increased by one nucleotide until a suitable simulant was identified or 10 window expansions had occurred, whichever happened first.

### Minigene constructs

A minigene construct for *ACP1* (ENST00000272065) was ordered from GeneArt as a double-stranded DNA string subcloned into the Gateway-entry vector *pENTR221*. The minigene consisted of the 5′ flanking exon, 5′ flanking intron, focal exon, 3′ flanking intron and 3′ flanking exon (see Supplementary Spreadsheet S5 for sequence information). Two versions were designed: one in which the wild-type sequence of the focal exon is preserved (‘wt’) and one containing the PTC-causing mutation (‘PTC’). To allow these genes to be translated, a start codon (ATG) was added at the 5′ end of all sequences. The 3′ flanking exon is the final exon and therefore already contains a stop codon (TGA). All minigenes were subcloned into *pCM3*, a Gateway-compatible CMV-driven mammalian expression vector (described in (83)), using Gateway LR Clonase II enzyme mix (Thermo Fisher) according to manufacturer's instructions. *pCM3* additionally also drives the constitutive expression of *mKate2* from an independent expression cassette which allows to correct for technical variability in transfection efficiency. The control NMD reporter constructs of human *TCR*-β have been previously described (84).

### Plasmid and siRNA transfections

HeLa and Hek293T cells were maintained in DMEM (Gibco) supplemented with 10% fetal calf serum (FCS) at 37°C, 5% CO_2_. NMD knockdown experiments were performed by two rounds of consecutive transfections with siRNA targeting *Upf1* (sihUPF1-I: GAGAAUCGCCUACUUCACU (+UU) and sihUPF1-II: GAUGCAGUUCCGCUCCAUU (+UU), Dharmacon, mixed in equimolar ratio). As a negative control, cells were transfected with a non-targeting control siRNA (ON-TARGETplus Non-targeting Control Pool, Dharmacon). In brief, cells were grown to 40% confluency in 12-well plates before transfecting with 1.25 μl of 20 μM siRNA stocks using 5μl Dharmafect1 transfection reagent (Dharmacon). After 48 h, the siRNA transfection was repeated using Lipofectamine 2000 transfection reagent instead (Thermo Fisher) and with the addition of 100ng of *pCM3* plasmid carrying the minigenes. Cells were grown for a further 48hrs before harvesting.

### RNA extraction and RT-PCR analysis

RNA from transfected cells (3 biological replicates for each condition) was extracted using the Qiagen RNAeasy kit according to manufacturer's instructions, including the on-column DNase I digest step. cDNA synthesis was performed using SuperScript III Reverse Transcriptase (Thermo Fisher) with 1μg of RNA and using 500ng anchored oligo(dT)20 primers (Thermo Fisher). cDNA was further treated with 5U RNAse H (NEB) before diluting with 30μl nuclease-free water. 2μl of each cDNA dilution were used as template in PCR reactions using either AccuPrime *Pfx* DNA polymerase (Life Technologies; *ACP1* and *mKate2* for HeLa samples) or *Taq* DNA polymerase (Life Technologies; *ACP1* and *mKate2* for Hek293T samples) following manufacturer's recommendations and 0.3 μM of gene-specific primers (for primer sequences see Supplementary Spreadsheet S6), ensuring amplification is within the exponential range. For quantitative Real-time PCR measurements of *Upf1* and *TCR* expression, samples were analysed in triplicate reactions on a Roche LightCycler480 using Roche LightCycler480 SYBR Green I Master Mix. Relative expression levels were determined using the Comparative Ct method (85) and normalised against *GAPDH* levels. *ACP1* and *mKate2* PCR products were resolved on 1.5% agarose in TBE gels stained with Ethidium bromide and imaged on a Syngene U:Genius 3 gel imager. Bands were quantified via densitometry with background subtraction using Image Studio Lite (v5.2). The resulting signals from *ACP1* bands were further normalised to the signal of *mKate2* bands from the same respective cDNA to account for technical variability in transfection efficiency. PSI was calculated as before, using the normalised signal of full-length transcript divided by the normalised signal of full-length transcript plus the normalized signal of transcript with skipped exon. Relative exon skipping was calculated by dividing the normalised signal of transcript with skipped exon of any given condition by the normalised signal of transcript with skipped exon in the wt control (NTC) samples.

### Out of frame PTCs analysis

For the out of frame PTC analysis, when determining the SNP type (synonymous, missense, nonsense), we shifted the reading frame forwards and backwards by one nucleotide. As a result, if the three nucleotides starting from the shifted codon position encoded a stop codon, we called this a PTC. We then repeated the pipeline with shifted PTCs.

### ClinVar analyses

Disease-associated mutations were downloaded from the ClinVar database (https://www.ncbi.nlm.nih.gov/clinvar/, last accessed May 11 2018; (86)) and intersected with the filtered exon set to leave only SNPs that occurred in our coding exons (N = 156,730). We then verified the status of the disease-associated mutations, retaining only those labelled ‘pathogenic’ or ‘likely-pathogenic’ (although note that this classification is at the discretion of the submitters and hence not standardised). The mutation status of each SNP was then verified. *N* = 23 092 synonymous and nonsynonymous variants were retained for ensuring these variants were not used in the reference allele-matched simulations and for determining the exons in which they reside for exon comparisons. Nonsense mutations were intersected with the 1000 Genomes dataset and only the *N* = 7429 non-overlapping nonsense variants retained. This results in *N* = 6354 ‘pathogenic’ and *N* = 1075 ‘likely pathogenic’ variants.

### Splice variant prediction

PTC variants were analysed using MMsplice (72), a neural network model trained on large-scale genomics datasets to predict the effects of variants on exon skipping, splice site choice, splicing efficiency and pathogenicity. Variants were compiled into a single VCF file with effects predicted using the model default parameters (exon_cut_l = 0, exon_cut_r = 0, acceptor_intron_cut = 6, donor_intron_cut = 6, acceptor_intron_len = 50, acceptor_exon_len = 3, donor_exon_len = 5, donor_intron_len = 13, split_seq = False). Changes in exon inclusion are reported as mmsplice_dlogitPsi values, with negative values indicating a predicted increase in exon skipping (lower PSI) and positive values indicating a predicted decrease in exon skipping (greater PSI) due to the variant.

### Expression analysis

We used FANTOM5 data (87) to estimate expression parameters independently of the Geuvadis RNA-seq data that was used to analyse splice isoforms. We retrieved the phase 1 and 2 combined normalized .osc file from the FANTOM5 website (http://fantom.gsc.riken.jp/5/datafiles; last accessed 11 February 2016). We only retained samples where the name contained the string adult, pool1. All brain tissues except for the full brain sample and the retinal sample were removed to avoid redundancy. For each gene included in our analysis, we defined a region of 1001 base pairs centred on the start coordinate of the Ensembl transcript annotation as the promoter and associated all peaks that overlapped that promoter to that peak. If several peaks were associated to a single transcript, we summed the transcripts per million (TPM) within each sample across the peaks. A gene was considered to be expressed in a given tissue if TPM > 5.

## RESULTS

### Evidence that ESEs should be hotspots for disruptive nonsense mutations

Prior to examining predictions of the motif model, we start by asking whether ESEs are *a priori* likely to be hotspots for nonsense mutations that would disrupt splicing (i.e. break an ESE). As we previously noted, like other exonic motifs ESEs have a dearth of the trinucleotides TGA, TAA and TAG (40). Importantly, like these three codons, ESEs are also purine rich (52). These two features place ESEs in an unusual position in hexameric sequence space: they are expected to have a high rate of gain of nonsense mutations (owing to purine richness), while the same nonsense mutations are likely to destroy the ESE (owing to the rarity of nonsense mutations in CDS based motifs).

To consider this hypothesis more formally, we consider the INT3 set of 84 hexamers, this being a set of ESE hexamers found in at least three of four large scale surveys (52). We consider all possible mutations at all possible sites within the hexamers asking whether the mutation would generate a stop codon where there was none before (see Materials and Methods). There are 219 such nonsense mutations within the 84 INT3 ESEs. We next checked whether the new nonsense containing hexamer is a known INT3 hexamer. For example, TCAAGA→TGAAGA reflects an ESE transitioning to another ESE via a nonsense mutation, both hexamers featuring in the INT3 set. After elimination of all such instances 204 instances remain where an ESE becomes non-ESE (or at least aren’t in the INT3 set) owing to a nonsense mutation.

To determine whether 204 is an unusually high number, we repeated the same analysis but this time with 10 000 data sets of 84 pseudo-ESEs as the input data set. As we are interested in the hypothesis that ESEs have a higher rate of being broken by nonsense mutations than random sequences within CDS, we randomly selected 84 non-redundant hexameric sequences from CDS of human RefSeq genes. For each set of 84 hexameric pseudo-ESEs we then apply the same protocol as above, this time asking whether the nonsense containing mutated pseudo-ESE also features in the relevant pseudo-ESE hexameric list. From 10 000 simulations we find no simulation that has 204 or more nonsense mutations that move the hexamer from the pseudo ESE list to the non-pseudo ESE list (mean in randomized set = 131 ± 11.4 SD, max = 184). We conclude that compared to random CDS, ESEs are indeed especially prone to being the site of a nonsense mutation that breaks the ESE (from the above simulation, *P* < 0.0001).

To check that in part the result is owing to purine enrichment, for each hexamer in the INT3 list we randomly extracted a hexamer from the concatenated CDS that had the same total purine content and that wasn’t in the set of INT3 hexamers. We thus generated 10 000 random sets of 84 exactly purine matched randomised sets. Repeating the analysis, we now find that the real ESEs still have more nonsense mutations (*P* = 0.004), but not by as great a difference as before (for purine matched randomised sets mean = 170, ± 9.9 sd, max = 208). The purine matched set has significantly more opportunities for nonsense mutations than random CDS hexamers (from simulation, *P* < 0.0001), as would be expected given the purine richness of stop codons.

Part of the reason for the non-equivalence between the purine matched set and INT3 in nonsense mutation rate is likely to be a difference in stop codon density. Every instance of a stop codon in any frame in a hexamer represents a codon where a nonsense mutation cannot happen by definition and, in addition, increases the chances that a nonsense mutation in a different hexamer within the defined list generates a hexamer in the same defined list. In the INT3 set of 84 × 4 = 336 full codons only nine are stop codons (2.7%). By contrast, even in the purine matched controls extracted from CDS, of the 10 000 84 pseudo-ESE sets 87% have more than nine stop codons (mean = 13 ± 3.12 sd). Indeed, if instead of purine matching, we randomize the order of nucleotides in each hexamer (generating 10 000 sets of 84 shuffled hexamers in which each real hexamer is randomised once), then 98% of these sets have more stop codons than INT3 (mean = 15, ±2.8 sd), emphasising just how lacking in stop codons INT3 is. Considering the 596 purine-matched sets with exactly nine stop codons in the 336 full codons we find these sets to be closer to the INT3 set than the purine matched set (mean = 175 ± 8.43). That they still have fewer opportunities for nonsense mutations than INT3 possibly reflects the skewed usage of A over G within the purines and low T density (in INT3, A = 46.6%, C = 11.7%, T = 9.9%, G = 31.7%). Indeed, considering the 119 hexameric sets of shuffled ESEs that also have nine stop codons in the 336 possible codons (hence exactly the same nucleotide content and the same stop codon density as INT3), now we see no significant difference between INT3 and the randomised set (from randomisation, *P* = 0.08, mean = 190, ±8.7, sd). Thus, the low stop codon density and unusual nucleotide content, in part reflected as skewed purine content, can in large part account for INT3 being especially prone to nonsense mutations that disrupt ESE functionality.

### Evidence that only a minority of PTCs are associated with exon skipping

To address the predictions related to the commonality of PTC-associated splice disruption and to establish a set of PTCs that likely are (or are not) associated with NAS, we started by assembling a set of 541 PTC-containing exons in which we could quantify splicing (see Material and Methods, for summary of the 541 see Supplementary data S1). For each exon, the median percentage spliced in (PSI) was calculated (see Materials and Methods) for each of the three genotypes: homozygous non-PTC (PTC−/−), heterozygous PTC (PTC-/+) and homozygous PTC (PTC+/+). We focus on comparisons between PTC−/− and PTC−/+ variants as only 24/541 (4.37%) exons had an individual with a PTC +/+ variant.

**Figure 1.**
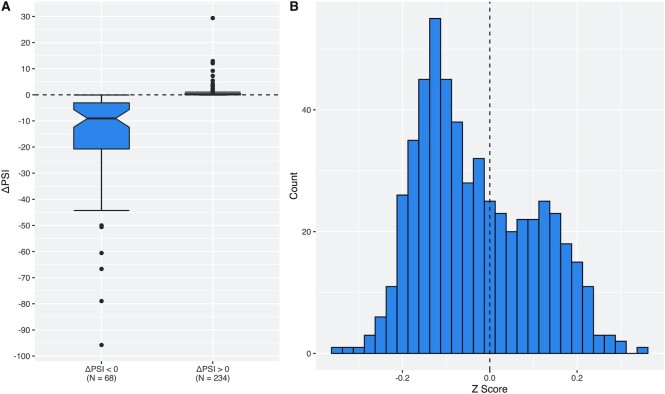
Differences in relative exon skipping levels. (**A**) ΔPSI scores for exons with non-zero differences in PSI between the two genotypes. ΔPSI scores corresponding to exons for which the PTC is associated with increased exon inclusion (ΔPSI > 0) are typically small. For distribution see Supplementary Figure S2A. (**B**) *Z* scores comparing the ΔPSI score for each PTC with ΔPSI scores for 100 missense mutations matched by ancestral allele, variant allele, allele frequency and distance to exon boundary.

We first asked whether there are detectable differences in exon inclusion for the same exon as a function of PTC presence. If PTCs are responsible for exon skipping, we expect the PSI for PTC−/+ variants to be lower than for PTC−/− variants. This is also quite a generous test as we employ exons annotated as constitutively included. Wild-type PSI is thus expected to be close to 1 in the majority of cases. As a consequence, when the PSI of the PTC−/+ genotype differs from that of the PTC−/−, it is more likely to be decreased than increased, simply because PSI cannot exceed 1. We find that PTC−/+ variants indeed have significantly lower PSI than PTC−/− variants, although the difference is small (mean PSI ≈ 0.960 and ≈ 0.977, respectively; *P* = 1.056 × 10^–5^, two-tailed paired *t*-test) (see Figure 1A and Supplementary Figure S2A). Almost half of the exons exhibit no difference in PSI between the genotypes (ΔPSI = 0, *N* = 239, 44.18%). In the majority of cases, therefore, the presence of a PTC appears to have little to no effect on skipping. However, a minority of cases do show large, several-fold changes in the exclusion level of the exon associated with the presence of a PTC (Figure 1A, left side in the histogram). This is consistent with the motif disruption model of NAS, which predicts only a subset of PTCs to lead to exon skipping.

### PTCs are associated with effects beyond what is expected given their nucleotide composition

Given that even by chance alone we expect a bias to negative ΔPSI values due to the boundary at PSI = 1, we asked whether nonsense mutations specifically are associated to particularly large increases in exon skipping when compared to other mutations. To account for nucleotide biases associated with mutations generating PTCs (see Supplementary Text S1), we selected a control set of missense mutations of similar nucleotide composition for comparison.

Specifically, for each PTC we simulated 100 pseudo-PTCs (pPTCs) by randomly sampling missense mutations across the genome matched by ancestral allele, variant allele and variant allele frequency (e.g. if the total PTC count on both alleles was 6/300, the matched mutation allele frequency was ≈0.2). To quantify any difference in ΔPSI, we calculated a *Z* score for each PTC, defined as the ΔPSI for the true PTC minus the mean of pseudo-ΔPSIs (ΔpPSI), divided by the standard deviation of ΔpPSI. Thus, if real PTCs have a more negative effect on PSI than the matched pPTCs as a result of being a PTC and not the nucleotides involved, *Z* scores will be negative.

We find 308/541 (56.93%) PTCs have a Z score less than zero, a small but significant deviation from null (*P* = 7.213 × 10^–4^, one-tailed exact binomial test, Figure 1B). This suggests that nonsense mutations are more likely to be associated with exon skipping than comparable mutations that are not nonsense mutations. We can also control for position by sampling a matched mutation from within the 10 bp window around true nonsense variant location (342/541 exons with *Z* < 0, *P* = 4.125 × 10^–10^, one-tailed exact binomial test). Collectively these results imply that nonsense mutations are special as regards exon skipping. These results also suggest that the bounding of PSI between 0 and 1 is unlikely to explain entirely the association between PTCs and exon skipping.

### NMD cannot account for many cases of increased exon skipping associated with a PTC

A possible alternative explanation for the association between PTCs and exon skipping is NMD, as it would remove full length isoforms with the PTC, causing an increased proportion of skipped reads, when in practice there has been no change in the absolute skipping rate (see Supplementary Text S2). It is therefore necessary to eliminate any PSI variations we observe that are also consistent with NMD. To do this, we used the absolute read counts supporting exon skipping or inclusion, normalised to the number of total reads per million to control for differing read depths between samples (RPMskip and RPMinclude, see Materials and Methods).

**Figure 2.**
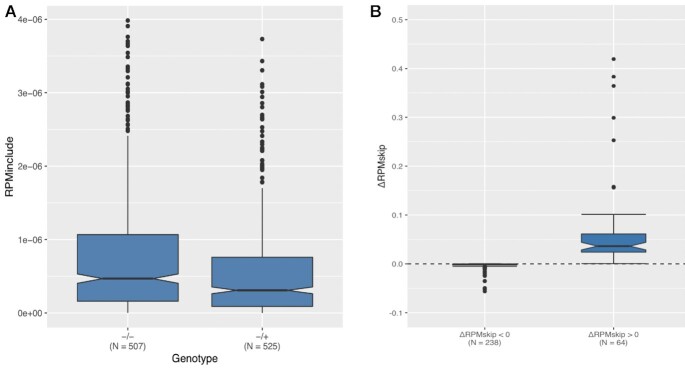
Differences in absolute skipping levels. (**A**) Raw read counts per million reads supporting exon inclusion (RPMinclude) for non-PTC and PTC variants. 50 outlier data points are removed for visualisation purposes. (**B**) Non-zero ΔRPMskip scores are consistent with those in the direction consistent with NAS having larger effects. The median negative ΔRPMskip is −4.794 × 10^–4^ arguing that when the PTC is associated with reduced exon skipping, the effect is almost negligible. One data point for ΔRPMskip < 0 at *y* = −0.759 and two outlier data points at for ΔRPMskip > 0 at *y* = 1.242, *y* = 4.778 are omitted for visualisation purposes. For distribution see also Supplementary Figure S2B.

We first asked whether we could detect NMD. If so, the raw number of reads supporting inclusion, RPMinclude, should be significantly higher in PTC−/− than for PTC-/+ variants, i.e. ΔRPMincl < 0 as full-length transcripts containing a PTC are likely to be subject to NMD at some rate. Our results suggest this is the case (*P* ≈ 2.648 × 10^–7^, one-tailed paired t-test, Figure 2A), with the median RPMinclude for PTC−/+ variants (0.321) almost one third less than the median RPMinclude for PTC−/− variants (0.508).

If PTCs are associated with exon skipping, RPMskip should be greater for PTC−/+ than for PTC−/− variants. PTC−/+ variants indeed have significantly higher RPMskip values (*P* ≈ 0.019, one-tailed paired *t*-test; Figure 2B; Supplementary Figure S2B), however, as with PSI, many cases have ΔRPMskip = 0 (*N* = 239). Is the number of PTCs with raw read counts supporting greater skipping for the PTC variant also higher than expected given the nucleotide composition of PTC mutations? We reanalysed the set of 100 matched missense simulants and asked how many PTCs differ in ΔRPMskip when compared with simulant pseudo-ΔRPMskip (ΔpRPMskip) values. A significant number, 339/541 (62.66%), have positive *Z* scores (*P* = 0.004, one-tailed exact binomial test; note here a positive *Z* score indicates increases in RPMskip over the simulants). This result is robust to missense mutations being matched by their distance to the exon boundary (381/557, *P* < 2.2 × 10^–16^, one-tailed exact binomial test).

These results suggest that the association between PTCs and exon skipping cannot be explained solely by NMD, as NMD should not affect the absolute count of reads that support skipping.

### Evidence that 6% of nonsense mutations may result in exon skipping

The above results provide, to the best of our knowledge, the first evidence of genome-wide associations between PTCs and exon skipping. However, in most cases the effects are very small and thus the change in PSI observed may not be biologically meaningful, in the sense that we may just be witnessing experimental noise (see the clustering of values around 0 in Supplementary Figure S2A). How frequently is NAS associated with changes in PSI that are large enough that they could be biologically meaningful?

**Figure 3.**
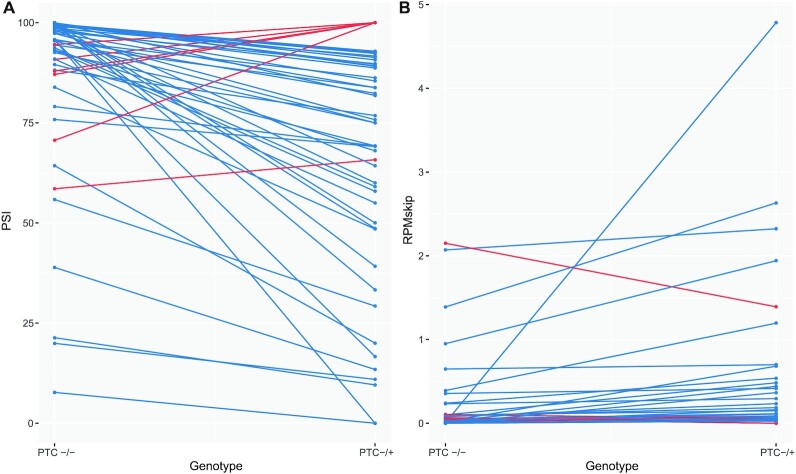
Individual large effect cases for both PSI and RPMskip. Large differences between PTC−/− and PTC−/+ genotypes for (**A**) PSI and (**B**) RPMskip. Variants with changes between genotypes consistent with NAS are in blue, those in the opposite direction in red. For both PSI and RPMskip, the number of large-effect variants in the direction consistent with NAS is significantly greater than expected by chance.

While setting a threshold is to some degree arbitrary, we suggest that a variant with a PSI > 5% or a change in RPMskip > 0.026 (see Supplementary Text S3) will be unlikely to be owing to noise and indicative of some meaningful biology. A 5% figure is not entirely arbitrary as it is both close to a turning point for statistical significance and accords with recommended cut-offs when employing similar data (88) (for fuller consideration see Supplementary text S3 and Supplementary Figure S3). With such cut-offs, 50/541 (9.24%) exons show a large effect change in PSI, of which 44 (88.00%) have ΔPSI < 0 with many far exceeding the 5% threshold Figure 3A). The direction of this enrichment is highly significant (*P* = 1.662 × 10^–8^, one-tailed exact binomial test). For RPMskip, we find 43/50 (86.00%) large-effect cases have ΔRPMskip > 0, indicating increased exon skipping for the PTC−/+ variant (Figure 3B), significantly higher than expected by chance (*P* = 1.049 × 10^–7^, one-tailed exact binomial test).

However, with caveats to both PSI (NMD effects) and RPMskip (possible effects of differing transcript abundances in the two differing genotypes) metrics, the most robust candidate exons with meaningful NAS-associated biology are those overlapping both large-effect ΔPSI and large-effect ΔRPMskip groups. 30 of the exons that appear in both groupings show more exon skipping in the PTC−/+ genotype (Table 1). To ask whether this overlap is significant, we performed 10,000 simulations picking 44 and 43 exons from the full set of PTC-containing exons (44 and 43 correspond to the number of exons with large NAS-consistent effect sizes for PSI and RPMskip respectively). We find no simulation iteration has an overlap as large as the real overlap (*P* ≈ 9.999 × 10^–5^, one-tailed empirical *P*-value, maximum simulant overlap = 10).

**Table 1.** 30 prime NAS candidates

Exon ID	PSI−/+	PTC−/−	ΔPSI	RPMskip−/+	RPMskip−/−	ΔRPMskip	ΔlogitΨ
ENST00000272065.5	39.19	99.76	−60.57	4.784	0.007	4.777	−0.520
ENST00000325083.24	29.26	55.84	−26.59	2.631	1.389	1.242	−0.266
ENST00000271324.6	88.69	98.20	−9.51	1.195	0.391	0.804	−0.166
ENST00000400033.8	16.67	95.62	−78.96	0.681	0.022	0.659	−0.963
ENST00000216027.4	59.09	93.71	−34.62	0.483	0.100	0.383	−0.432
ENST00000359028.47	64.29	99.83	−35.55	0.366	0.001	0.364	−0.465
**ENST00000367409.18**	**69.08**	**75.82**	**−6.74**	**0.538**	**0.239**	**0.299**	**0.005**
ENST00000267430.22	48.63	99.24	−50.61	0.162	0.004	0.158	0.560
ENST00000288050.18	76.81	88.17	−11.36	0.234	0.078	0.156	−0.163
**ENST00000456763.12**	**75.76**	**97.33**	**−21.57**	**0.111**	**0.011**	**0.100**	**−0.029**
ENST00000255409.8	57.89	93.08	−35.18	0.111	0.018	0.093	−0.144
ENST00000272252.4	89.09	99.88	−10.79	0.079	0.001	0.078	−4.478
ENST00000222800.4	68.00	93.15	−25.15	0.110	0.032	0.078	−0.090
ENST00000382977.11	33.33	100.00	−66.67	0.073	0.000	0.073	−0.804
ENST00000389175.23	20.00	64.30	−44.30	0.113	0.052	0.061	−0.151
**ENST00000265316.3**	**83.78**	**97.45**	**−13.67**	**0.079**	**0.018**	**0.061**	**0.053**
ENST00000355774.3	89.19	99.93	−10.75	0.054	0.000	0.054	−0.379
ENST00000398141.8	10.95	19.95	−9.00	0.699	0.648	0.052	−0.640
ENST00000357115.15	90.70	99.74	−9.04	0.053	0.003	0.051	−0.570
**ENST00000487270.3**	**92.59**	**99.68**	**−7.08**	**0.052**	**0.002**	**0.050**	**−0.251**
ENST00000216294.2	92.31	99.55	−7.24	0.054	0.003	0.050	−0.150
ENST00000338382.7	91.30	99.77	−8.47	0.053	0.003	0.050	−0.559
ENST00000331493.9	9.58	21.35	−11.77	0.149	0.099	0.050	−0.203
ENST00000328867.14	69.23	89.54	−20.31	0.055	0.014	0.041	−1.237
ENST00000376811.6	89.58	99.50	−9.91	0.041	0.003	0.037	−0.092
ENST00000535273.7	83.78	94.41	−10.62	0.081	0.045	0.036	0.116
**ENST00000370132.6**	**85.45**	**98.61**	**−13.16**	**0.041**	**0.008**	**0.033**	**−0.221**
ENST00000542534.16	50.00	100.00	−50.00	0.027	0.000	0.027	−0.167
ENST00000354366.10	81.82	99.98	−18.16	0.027	0.000	0.027	−0.153
ENST00000238561.9	82.35	95.81	−13.45	0.041	0.015	0.026	0.018

The 30 prime NAS candidates are those supporting an association between the PTC and increased relative exon skipping (ΔPSI < –5), as well as absolute exon skipping (ΔRPMskip > 0.026), sorted by decreasing ΔRPMskip. Exon ID is defined as ‘ensembl_transcript_id.exon_number’ where the exon number is incremented in the direction of transcription. ΔlogitΨ scores are those predicted by MMSplice. PTCs that also appear in the ClinVar dataset are shown in bold.

These PTCs are prime candidates for cases in which a single nucleotide polymorphism (SNP) generating a PTC causes potentially detrimental exon skipping via NAS. We estimate that it is possible ≈ 6% (30/541) of annotated PTC mutations cause NAS (these are annotated as ‘primes’ in Supplementary data S1). Results using an alternative data source produce a similar estimate (≈4%, Supplementary Text S4), however we caution that this result is underpowered.

Aside from the splicing characteristics, little differentiates these 30 from the 511 remaining cases: there is no difference in the size of the exon (*t* test, log exon size, *P* = 0.51) nor in the distance of the stop codon from the nearest exon intron boundary (*P* ∼ 0.429 from a two-tailed Welch's *t*-test: Supplementary Figure S4).

We also examined the rarer PTC+/+ instances (24/541). Results are similar to those for PTC+/− variants, with PSI values (median negative ΔPSI = −11.920, median positive ΔPSI = 0.191) and RPMskip (median negative ΔRPMskip = −0.001, median positive ΔRPMskip = 0.261) in the direction consistent with NAS. However, given the limited sample size we again caution against over-interpreting this result. Note that all exons that had individuals with a +/+ genotype also had individuals with a +/− genotype. Therefore, our decision not to include PTC homozygotes into other analyses did not lead to the exclusion of any exons, just to the exclusion of some of the samples for these exons.

### No evidence for *cis* effects

It is possible that some PTCs are not causative of the splice disruption that we observe but other *cis*-mutations in the same exon are instead. This, we suggest, will have a very minor relative effect on rate estimation, if any, as, of our 30 PTC candidates, 28 have no other SNP in the same exon as that bearing the PTC. Moreover, of the other two (ENST00000367409.18, ENST00000542534.16), the SNPs identified were all common within 1000 Genomes data indicating that it is unlikely that they are causative of the splicing effect. ENST00000542534.16, for example, has one SNP (15:42135988:C:T) in the exon that also bears the PTC but this SNP is at a frequency of 76%. Were this causative, we should have seen skipping at much higher rates. Altering the thresholds for PTC calling will thus most probably provide significantly more influence over the estimate of the lower bounds than removal of exonic *cis* effects.

### Experimental validation of the top NAS candidate

Having computationally identified potential NAS candidates, we sought to validate our results experimentally. Here, we do not intend to provide evidence as to the mechanism, just to confirm that there is NMD-independent NAS, as predicted by our bioinformatics pipeline.

**Figure 4.**
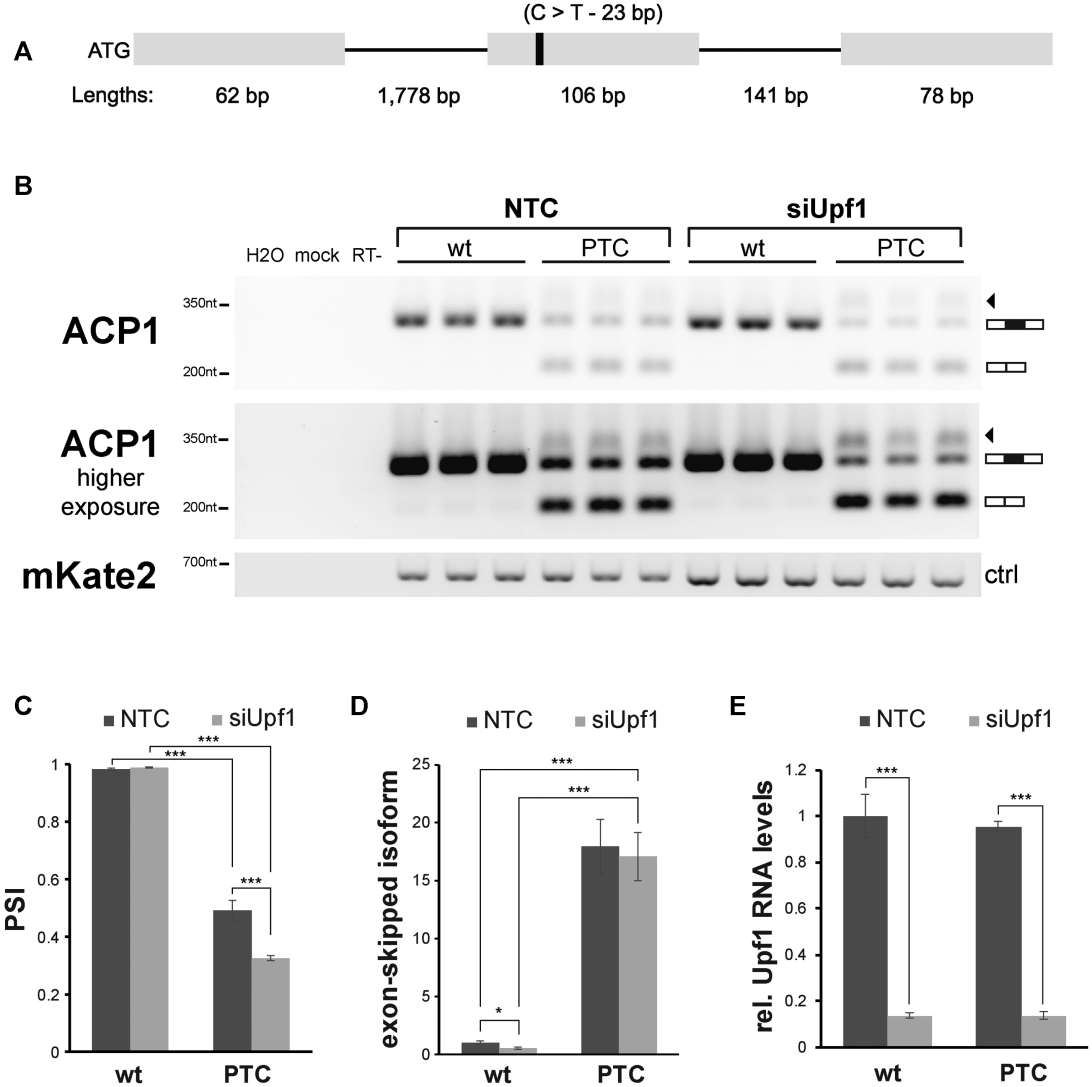
Experimental validation of the top NAS candidate located in the *ACP1* gene. (**A**) Schematic overview of the minigene construct. Exon/intron lengths are defined below the relative minigene section, with ATG appended to the minigene 5′ terminal to allow for protein translation. The variable site, position 23 of exon 2 (solid black bar), contained either a C (wt) or T (PTC variant). (**B**) Agarose gel electrophoresis of RT-PCR of HeLa cells (three biological replicates each) treated with either a non-targeting siRNA pool control (NTC) or Upf1-targeting siRNA (siUpf1). mKate2 levels are shown as transfection control. (**C**) PSI levels for wt and PTC-containing ACP1 variants. Bands corresponding to full-length transcript and transcript with skipped exon were first normalised to mKate2 before calculating PSI as full-length transcript divided by full-length transcript + transcript with skipped exon (*n* = 3). (**D**) Relative levels of exon skipping are shown as the ratio of normalised levels of transcript with skipped exon in a given condition to the normalised levels of transcript with skipped exon in the wt NTC control (*n* = 3). (**E**) Relative Upf1 mRNA expression levels in Upf1 knockdown and control cells. Error bars denote the standard error of the mean for 3 biological replicates. Tests are two sample t-tests.

A minigene construct for the prime candidate exon from the *ACP1* gene with the greatest ΔRPMskip (ENST00000272065.5, Table 1) was constructed and expressed as described in the Materials and Methods (Figure 4B). In HeLa cells, we find a significant difference in PSI between the wt and PTC-containing constructs (*P* = 2.226 × 10^–5^, two sample *t*-test, Figure 4A and B), with skipping almost exclusively restricted to the PTC-containing construct. Consistent with skipping resulting from NAS and not NMD, this difference in PSI remains after knockdown of the core NMD factor Upf1 (*P* = 2.783 × 10^–8^, two sample *t*-test, Figure 4B and C). As a side note, the isoform with the PTC-containing exon is not a significant target of NMD; if it were so, we would expect the isoform to be more abundant in siUpf1 cells compared to control cells, while we observe the isoform to be marginally less abundant in the siUpf1 context (Figure 4B).

We also asked whether levels of the exon-skipped isoform significantly differ as expected were NAS the underlying cause. We find an increase in levels of the skipped isoform with inclusion of the PTC (*P* = 1.804 × 10^–4^, two sample *t*-test, Figure 4D) and again when NMD is knocked down (*P* = 2.741 × 10^–4^, two sample *t*-Test, Figure 4D), suggesting that the presence of the PTC results in an increase in the absolute number of transcripts supporting skipping regardless of NMD. Consistent with this notion, skipping for PTC variants does not significantly differ between cells where NMD is present or knocked down (*P* = 0.302, two sample *t*-test, Figure 4D). We infer that NMD cannot explain the decreased exon inclusion associated with the PTC. To confirm that NMD was depleted, levels of Upf1 mRNA were quantified. We find that Upf1 mRNA levels are significantly lower in the Upf1 knockdowns compared with control cells (*P* < 0.001, two sample *t*-tests, Figure 4E), as well as confirm that NMD is functionally depleted using TCR-beta reporter constructs (Supplementary Figure S5C) (84). We also note that experimental PSI and skipped isoform levels are broadly consistent with our computational PSI calculations for the 1000 Genomes samples. We find similar patterns in Hek293T cells (Supplementary Figure S5) thus demonstrating PTC-associated exon skipping independent of cell type. For full gel images underlying Figure 4, see Supplementary Figure S6, for those associated with Supplementary Figure S5 see Supplementary Figure S7.

The above validates that our bioinformatic pipeline's top hit is a bona fide example of NMD-independent NAS. While here we are not concerned with the mechanism in this instance, we suggest that the minigene construct recapitulating what is seen in vivo, provides good raw material for downstream analysis.

We also sometimes observe a faint third band for the PTC mutants (Figure 4B, black triangle). However, this is not seen in both cell types (Supplementary Figure S5) and failed attempts at replication. We therefore consider it unsafe to draw any inference from the presence of this band.

### Large-effect PTCs are *in silico* predicted to have larger increases in exon skipping when compared with the other PTCs

The above analysis provides lower bound estimates for the frequency of PTC-mediated alternative splicing (ca. 4–6%). As our bounds for inclusion (5% difference) are relatively weak, this may be considered a generous lower bound, but as such also suggests that NAS is typically not induced by polymorphic PTCs. We consider upper bound estimates below when considering ClinVar data. First, however, we further analyse the 1000 Genomes data to address the further two predictions of the motif model.

If NAS is owing to motif disruption, then motif/nucleotide based inference models should be able to predict which PTCs are splice disruptive. Note that the machine learning models are not trained on stop codons. We predicted changes in PSI for each PTC using MMSplice (72), a neural network model that outperforms other splicing variant scoring models (HAL (89), SPANR (90) and the baseline predictor model MaxEntScan (91)). MMsplice reports the effect of a variant on PSI on the logistic scale (ΔlogitΨ), with ΔlogitΨ < 0 indicating a predicted increase in exon skipping associated with the variant. We find 25/30 (83.33%) of our large-effect candidates are predicted to increase skipping in this model, significantly more than expected by chance (*P* = 3.249 × 10^–4^, two-tailed exact binomial test). Further, these differences in predicted skipping are significantly greater than for the remaining 511 variants (median large-effect PTC ΔlogitΨ = −0.185, median other PTC ΔlogitΨ = −0.140, *P* = 0.0242, one-tailed Wilcoxon rank sum test).

While then NAS can be predicted by sophisticated machine learning methods to be predict splicing modification, we find no evidence that the 30 NAS associated mutations are any more likely to disrupt an ESE than the 511 not considered NAS candidates. Using the INT3 dataset, for example, and considering the nucleotides −5 to +5 of the focal mutation (i.e. allowing the mutation to be anywhere between the end or beginning of an ESE hexamer), we find no evidence that the 30 NAS candidates have a higher density of ESEs in this span than the 511 (Mann−Whitney *U* test, *P* = 0.39). Asking about the number of cases where one or more ESE is seen overlapping the focal mutational position, we see no difference between the NAS candidates (6 with a ESE, 24 without) and others (70 with an ESE, 441 without) (chi squared with Yates’ correction = 0.93, *P* = 0.33), although the frequency (20%) is higher for the former than the latter (13.7%). We also see no correlation between ΔPSI and the number of ESE motifs associated with any given span in the proximity of any mutation (spearman rank test, rho = 0.005, *P* = 0.90). We conclude that presence of ESEs at the site of the mutation is not a good predictor of which nonsense mutations do or do not induce NAS in this dataset. The candidate set (*N* = 30) and the remaining exons (*N* = 511) are no different in ESE density within an exonic compartment (i.e. 5′ flank ESE density of the NAS set is no different from 5′ flank density of the non NAS set etc, Supplementary Figure S8). Employing only those exons in which the core rate can be measured, we recover the classical result that exon flanks tend to have higher ESE density than exon cores (Supplementary Figure S8B).

### Out of frame mutations to TAA, TGA or TAG also are associated with exon skipping

To further scrutinize the motif model we analysed mutations to TGA, TAA or TAG that are out of frame by one nucleotide. These we refer to as mutations creating ‘shiftPTCs’. We retain only one mutation per exon and asked if they were also associated with exon skipping, as predicted by the motif model.

We performed similar analyses to those above. We find that the absolute differences in shiftPTC PSI scores between the variants (ΔshiftPSI) were significantly greater for differences consistent with NAS (*P* < 2.2 × 10^–16^, one-tailed Wilcoxon rank sum test, median ΔshiftPSI > 0 = 0.049, median ΔshiftPSI < 0 = −0.726). Further, we find the same is true for RPMskip for the shiftPTCs (ΔshiftRPMskip) (*P* < 2.2 × 10^–16^, one-tailed Wilcoxon rank sum test, median (ΔshiftRPMskip > 0 = 0.011, median (ΔshiftRPMskip < 0 = −3.950 × 10^–4^).

218/6948 exons exceed the large-effect PSI threshold (>5%), with a significantly greater number showing decreased PSI in the shiftPTC−/+ than for the shiftPTC−/− variants (171/218, *P* < 2.2 × 10^–16^, one-tailed exact binomial test, see Supplementary Figure S9A). Further, 183/218 (83.94%) cases have ΔshiftRPMskip > 0 consistent with NAS (using the threshold of 0.04871, this being the cut-off to size match the 218 PSI variants), a significant number (*P* < 2.2 × 10^–16^, one-tailed exact binomial test, see Supplementary Figure S9B). 100/218 (1.44% of total shiftPTCs) of these shifted PTCs have greater than 5% difference for both PSI and RPMskip. The number of large-effect cases is significantly lower than for those in-frame (χ^2^ = 47.231, *P* = 6.309 × 10^–12^, chi-squared test). However, simulations picking 171 and 183 cases at random suggest the 100 large-effect cases with both PSI and RPMskip in the direction consistent with NAS is more than expected by chance (*P* ≈ 9.999 × 10^–5^, one-tailed empirical *P*-value).

As out of frame mutations to TAA, TGA or TAG would not be subjected to NMD, the above result provides further evidence to suggest that NMD cannot explain all of the previous differences in exon inclusion we observe between genotypes.

### Enrichment analysis predicts that about a third of pathogenic nonsense mutations may have their effect via splicing

PTCs associated with disease phenotypes are expected to be enriched for cases of NAS and thus provide a means to estimate an upper bound for the rate at which PTCs disrupt splicing. Here our method is more indirect. We ask about the extent of end of exon enrichment of known disease-associated nonsense mutations (similar to (71)), as splice modifying mutations are enriched towards exons ends (49), where ESEs, other splice signals and splice disrupting mutations typically reside (48,49,52–54,92,93). We also ask whether the end-of-exon PTCs are likely to be splice-modulating by looking for ESE enrichment and via *in silico* prediction.

We assume exonic core pathogenic nonsense mutations (beyond both the 5′ and 3′ terminal 69 nucleotides) not to have a major effects on splicing (48). Their rate thus provides us with a background level (although is likely conservative as splice-affecting mutations also occur in exon cores (49)). Any excess of pathogenic nonsense mutations above this core level we then assume to be splice-related. This is also a strongly indicative metric as enrichment at exon ends isn’t obviously expected by alternative mechanistic models (NMD and protein truncation). Indeed, if anything, as NMD cannot detect some PTCs towards the end of the last but one exon (94), NMD based mechanisms might predict weak enrichment away from exon ends. Moreover, with lower SNP levels at exon ends (48) in healthy individuals, any enrichment in PTCs is unlikely to have a mutational explanation.

Using a set of disease-associated mutations from the ClinVar dataset (70), we find both ‘pathogenic’ and ‘likely pathogenic’ variants occur in exon flanking regions more frequently than expected by chance (see Supplementary Text S5). Taking the coding exons in which pathogenic nonsense mutations occur (N = 3,572), we define the exon core as any nucleotide beyond the terminal exon 69 nucleotides, this being the approximate upper range of ESE activity (48), although some ESEs show constraint past this (57) and a few are functional in exon cores (95). We observe 1,804 nonsense mutations within the 321 918 nucleotides of exon cores at a rate of 0.0056 mutations per nucleotide. Thus, assuming exon flanks behave like exon cores we expect ≈ 2365 nonsense mutations in the 422 017 exon flank nucleotides. Instead, we observe 4447, an excess of 2082 (32.77%) of mutations (see Supplementary Spreadsheet S4). This suggests that in regions with an increased density of splice information, pathogenic nonsense mutations occur much more frequently than expected. The effect of likely pathogenic mutations appears stronger with an excess of 58.49% of nonsense mutations in exon flanks (see Supplementary Spreadsheet S4).

The above estimate is higher than a more conservative estimate of 10% of all mutations that cause disease by splice disruption. As this required both *in vitro* and *in vivo* verification of splice disruption (67) this is to be expected. Our estimate is in line with enrichment analyses similar to ours that estimate a third of all disease-associated mutations to modulate splicing (62,71), but is higher than an early estimate of circa 15% (96) that considered only mutations in the immediate vicinity of splice sites (see also 97).

### Disease-associated PTCs are disproportionately embedded in ESEs

**Figure 5.**
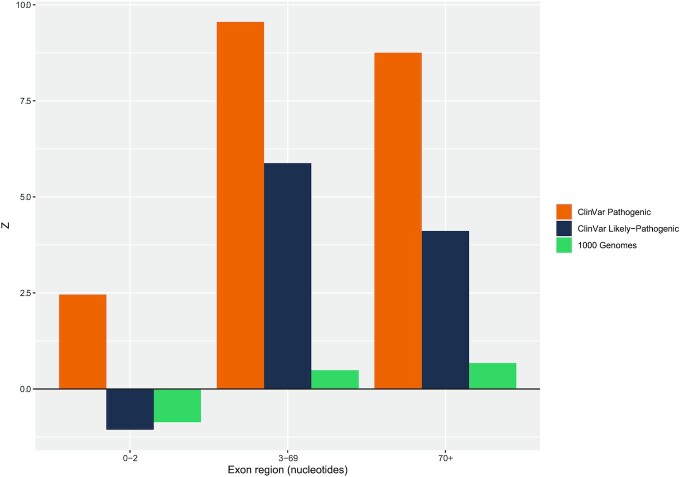
Frequencies of ESE nucleotides hit by PTCs in respective exon regions. *Z* scores comparing how frequently pathogenic and likely pathogenic variants from the ClinVar data set and variants in the 1000 Genomes dataset hit ESE motifs when compared with 10 000 randomly sampled nucleotides matching the reference-allele of the nonsense mutation SNP variant. Pathogenic and likely pathogenic variants hit ESEs significantly more frequently than expected (*P* ≈ 9.99 × 10^–5^ in both cases), although the enrichment over expected is stronger for pathogenic variants. Consistent with the non-exceptionality of the 1000 Genomes variants, these do not hit ESEs more frequently than expected in any region when compared with the randomly sampled variants.

Despite this biased ‘end of exon’ distribution, pathogenic nonsense mutations may not disrupt splicing. As the strongest signal of selection on splice motifs is on ESEs, as opposed to ESSs (55), we asked whether pathogenic mutations disrupt ESEs more than expected by chance. Specifically, we determined whether the pathogenic nonsense mutations hit one of the INT3 ESEs (52) in the exon flank regions more frequently than reference allele-matched simulants (N.B. INT3 is a low false positive set of ESE motifs observed in at least three of four systematic surveys). We find this to be the case (*Z* = 9.555, *P* ≈ 9.99 × 10^–5^, one-tailed empirical *P*-value, Figure 5). ‘Likely-pathogenic’ mutations also hit ESEs within the exon flanks more frequently than expected (*Z* = 5.877, *P* ≈ 9.99 × 10^–5^, one-tailed empirical *P*-value) although the effect is weaker than for the well-characterised pathogenic variants. The same is not seen for the PTC variants in 1000 Genomes data (above and Figure 5).

However, simulated mutations occur less frequently than true nonsense mutations in the 3–69 nucleotide region (see Supplementary Text S5). Therefore, for each pathogenic nonsense mutation in the 3–69 nucleotide exon region we randomly picked a nucleotide-matched pseudo-nonsense mutation (not generating a PTC in the ClinVar dataset) also from within the 3–69 nucleotide region. Again, the real nonsense disease-associated mutations hit ESEs more frequently than expected (*Z* = 1.920, *P* ≈ 0.030, one-tailed empirical *P*-value).

These results indicate that disease-associated nonsense mutations are distributed non-randomly in exons and hit ESEs more frequently than expected after control for mutational frequency (between exons and across the same exon), underlying nucleotide content of the 3–69 nucleotide region and relative expression (simulations are within the same exon). Moreover, pathogenic PTCs occur in non-3n exons more frequently when the exon is relatively long, with exons containing pathogenic PTCs typically having higher ESE density (Supplementary Text S6).

### *In silico* prediction supports a role for some disease-associated PTCs in splice modulation

Above we have considered one class of motif known to modulate splicing. We can also ask whether, more generally, machine learning approaches also predict splice disruption. We find that 82.75% (5258/6354) of variants had a negative effect on computationally predicted PSI, a significantly greater number than expected simply by chance (*P* < 2.2 × 10^–16^, one-tailed exact binomial test, null probability of success = 0.5). This effect is slightly more pronounced for variants occurring in exon flanks (3722/4447 (83.70%), *P* < 2.2 × 10^–16^, one-tailed exact binomial test, null probability of success = 0.5) but not significantly so (χ^2^ = 1.603, *P* = 0.206, chi-squared test), suggesting that pathogenic nonsense mutations in exon cores also frequently disrupt splicing, as described in minigene constructs (95).

The above result is, however, confounded by the fact that ‘short’ exons (those less than 138 bp) are all exon ‘flank’ in the sense that ESEs function up to ≈69 bp from an exon end (48). When restricting the analysis to only those exons longer than 138 bp, we find that although pathogenic nonsense mutations reduce PSI more than expected in both the exon flanks (1938/2327 (83.28%), *P* < 2.2 × 10^–16^, one-tailed exact binomial test, null probability of success = 0.5) and exon cores (1454/1804 (80.59%), *P* < 2.2 × 10^–16^, one-tailed exact binomial test, null probability of success = 0.5), the difference in the relative number of mutations decreasing PSI between the two regions is significant (χ^2^ = 4.805, *P* = 0.028, chi-squared test). Thus, our previous estimate of the number of pathogenic nonsense mutations disrupting splicing based on the core rate is likely conservative. We find a similar number of likely-pathogenic mutations decrease PSI (906/1075 = 84.28%), *P* < 2.2 × 10^–16^, one-tailed exact binomial test, null probability of success = 0.5).

Taken together with the excess in ESE flanking regions, it is reasonable to assume that splice disruption and exon skipping attributable to PTCs is likely to be a quite frequent source of pathogenicity.

## DISCUSSION

We considered the viability of the motif model to explain why PTCs can be associated with splicing disruption (NAS). *A priori* we reasoned that the motif model is parsimonious, not least because exonic regulatory splice motifs, such as ESEs, must and do, contain few stop codons (40). Exonic splice motifs may therefore be particularly sensitive to mutations creating TAA, TGA or TAG trinucleotides, including those out of frame.

The motif model makes a fairly robust account of the data. First, splice disruption associated with PTCs is not associated with all PTCs but just a limited subset (∼5% in non-disease data and ∼30% in disease associated PTCs). A midway estimate is roughly in agreement with analyses of random mutations (not PTCs) in minigene exons, which, after exon size correction suggests a similar figure for the proportion of mutations that disrupt splicing (58).

Second, the motif model predicts which subset of PTCs initiate NAS. We found that NAS presence/absence in 1000 Genomes data can be predicted from motif centred information, the machine learning motif recognition methods being trained predominantly in the absence of in-frame stop codons on splice patterns of exons. However, the skipping events seen in the 1000 genome data seem rather uncoupled from ESE mediated events suggesting that it is other motifs that the machine learning approaches identify. By contrast, enrichment of disease associated PTCs at exon ends and in ESEs is supportive both of the motif model and involvement of ESEs in particular. Third, that NAS is also seen in the out of frame context is supportive of the motif model.

### The relevance of the data for the scanning model

We have not considered the scanning model in detail and the sort of data that we analyse has rather little power to scrutinise it. It is not obvious that it makes predictions about the commonality of NAS (test 1). It also makes no strong claims (that we are aware of) regarding any motifs in the vicinity of the PTC that might induce scanning mediated NAS (test 2). That we find out of frame TAA, TGA and TAG mutations induce NAS would argue that some proportion of NAS cannot be explained by frame-dependent scanning (test 3).

While the data provide support for the motif model, they do not, however, falsify the scanning model. As the two models are not mutually exclusive, it is possible that some NAS is associated with scanning, for example for PTCs that aren’t embedded within splicing motifs and that are in frame. The fact that we see a difference between the rate of splicing disruption associated with in-frame nonsense mutations and out of frame TAA, TGA and TAG mutations is consistent with such a mixed model.

### Is NMD a confounding variable?

Can we be confident that NMD hasn’t interfered with analyses? The RPMskip data, analysis of out of frame stop codons and experimental data examining skipping in the absence of NMD, all argue against NMD as the cause of the effects that we see. It is also notable that without looking at the absolute ΔPSI values or correcting for nucleotide composition, we did not observe PTCs to associate with lower PSI. This is surprising given that such an association would be expected purely because of NMD downregulation of the full-length isoform, even if no NAS is occurring. One explanation could be that NMD is very weak in our samples. However, the large and highly significant decrease in RPMinclude in PTC−/+ samples argues against this scenario. Alternatively, it is possible that the mutations either create ESEs or disrupt ESSs leading to a slight increase in exon inclusion. A more likely explanation is that, at least for the exons being considered, splicing is very precise and, in most cases, no detectable exon skipping is observed. Indeed, the median PSI overall for PTC−/− samples is ≈ 99.994% and median ΔPSI ≈ 0.

### Are our estimates good upper and lower bound estimates?

Are we correct in thinking that our PTC/NAS rate estimates from ClinVar and 1000 Genomes data really represent upper and lower bounds? In both cases, the estimates either replicable (as we have shown) or comparable to prior similar estimates (see results). We also attempted to confirm the 4–6% lower bound using a third independent dataset (98) but this was not large enough to report meaningful information.

The lower bound estimate will be sensitive to the threshold employed to define NAS. As we make the cut-off more stringent, so naturally fewer PTCs would be classified as NAS associated (Figure 3A). However, while the 5% cut-off is somewhat arbitrary, many PTCs are associated with much stronger effects (Figure 3A). Whether it is 2% (more stringent) or 6% that are seen to affect splicing is rather irrelevant in the current context, as we are simply attempting to estimate lower bounds and these numbers are all to a first approximation congruent. The point of using a low threshold in the first place was to establish whether, with a possibly relaxed threshold, the great majority of PTCs are associated with splice disruption, which would not be consistent with the minigene experiments (58) after control for exon length effects.

We can also ask whether other evidence supports the presumed ascertainment biases affecting both estimates. One likely reason for the difference between the two estimates is a difference in selection acting on the two classes of PTC owing to sampling: under-estimating in the lower bound, over-estimating in the upper bound. As expected with such ascertainment biases, we witness a significant depletion in these flanking regions on nonsense mutations in the 1000 genome data, while in the ClinVar dataset we see a significant excess (see Supplementary Figure S10). These results are consistent with purifying selection acting especially strongly on mutations at exons ends (within 69 bp of the junction) and argues against a null of differential mutation rates across the exon. This strongly supports the notion that the two samples are likely to be affected by opposite ascertainment biases.

A further factor, aside for ascertainment biases, could be that our filtering process is likely to have excluded large-effect cases in genes that are lowly expressed (and therefore not considered due to few individuals with quantifiable splicing). We find no evidence to suggest that allele frequencies of the large effect variants are greater or less than for the other variants (*P* ≈ 0.200, one-tailed empirical *P*-value).

While we presume that our set of PTCs that are polymorphic in 1000 Genomes data are on average of lower fitness effects than those seen in ClinVar data, this need not be true for all 1000 Genomes PTCs. The literature contains several examples where our large-effect cases have been associated with disease. For example, the transcript with the largest PSI difference, ENST00000409520, is encoded by the *TraB domain containing 2A* (*TRABD2A*) gene associated with negative regulation of the Wnt signalling pathway, itself heavily implicated in cancers (99–105). Further, mutations in the *NDUFV2* gene producing the transcript ENST00000400033 have been associated with Parkinson's (106) and Leigh syndrome (107). Mutations in our prime candidate *ACP1* (ENST00000272065) have been associated with diabetes (108,109). Five cases also overlap with the disease-associated mutations in the ClinVar database (*rs62624965, rs202001274*, rs148458820, *rs200355697* and *rs74103423* (Table 1 bold, Table 2)). Whether NAS is the mode of operation in these instances we leave to future study.

**Table 2.** Further information regarding the five prime NAS candidates overlapping ClinVar variants

PTC ID	Exon ID	Mutation	Information
rs62624965	ENST00000367409.18	T > G	• *ASPM* gene.
			• Benign mutation (70).
			• *ASPM* produces two isoforms, one with exon 18 skipped, in both human and mouse and therefore may encode two proteins with different functions (133), thus skipping of exon 18 may not be as detrimental.
rs202001274	ENST00000456763.12	C > T	• *MAPKBP1* gene.
			• Associated with Nephronophthisis 20 (134).
			• Homozygous PTC Individual produced full-length and exon-skipped isoforms.
			• Thought to affect binding of serine-arginine rich (SR) protein SF2/ASF binding leading to exon skipped isoforms.
rs148458820	ENST00000265316.3	G > A	• *ABCB6* gene.
			• Mitochondrial porphyrin transporter essential for heme biosynthesis.
			• Associated with Langereis blood group (135).
			• May have implications in blood transfusions and drug therapies (136).
			• ABCB6 also thought to contribute to anticancer drug resistance (137).
rs200355697	ENST00000487270.3	C > T	• *RAD51B* gene.
			• Encodes a DNA repair protein.
			• Uncertain significance for hereditary cancer-predisposing syndrome.
			• *RAD51B* splice mutations leading to exon skipping have been associated with cancer (138).
rs74103423	ENST00000370132.6	G > T	• *Dihydrolipoamide branched chain transacylase E2* gene.
			• Associated with maple syrup urine disease (MSUD) (139).
			Truncated and exon skipped isoforms found.

Exon ID is defined as ‘ensembl_transcript_id.exon_number’ where the exon number is incremented in the direction of transcription.

### Skipping versus other modes of splice disruption

Exon skipping is the most common type of alternative splicing in wild type state in humans (66), in response to mutation (67) and associated with CRISPR generated indels (68,69) (many of which may be incidences of NAS). The commonality of skipping is extremely beneficial for this analysis as some modes of our analysis (transcriptomics) address skipping exclusively, while other modes (e.g. *k*-mer enrichment) are blind to the exact mode of splice disruption. As a consequence, to make the upper and lower bound estimates strictly comparable they would need to be rescaled. For example, as the lower bound 6% figure pertains to skipping alone, then to be directly comparable to the upper bound, and to the meta-analysis of minigene splicing disruption, this figure would need to be scaled up (or the other estimates scaled down). Given the relative commonality of skipping this we assume to be a relatively minor adjustment. Indeed, it probably brings the lower bound estimate in to line with the lower bound of the meta-analysis estimate, although given sensitivity to threshold cut-offs we ascribe little to this.

It is not unreasonable to suppose that nonsense mutations might act via modes other than exon skipping. Splice site creation may indeed, a priori, be a possible mode of action. The 5′ and 3′ splice site consensus motif AG|GT of U2 introns could be generated from a nonsense mutation of the sequence TA[C\T]GT via a C or T to G mutation at site three. Further, G|A can define rarely used U12 splice sites and hence appear as [C\G\A]GA→TGA mutations. Other modes are imaginable. A nonsense mutation could, for example, create an exonic splicing silencer (ESS) (a *cis*-regulatory element that inhibits the use of adjacent splice sites (110)) or modulate RNA structure (15), which may in turn modulate motif accessibility. One reason we chose not to consider ESSs was owing to a lack of certainty concerning their identity. In particular, prior analyses (55) detected no evidence for selection operating on the candidate (110) ESS motifs when in exons. The motifs also don’t show avoidance of stop codons, despite CDS exonic motifs being motifs that should (by definition) have low stop codon density (40,50).

### NAS is unlikely to be an evolutionarily conserved error-proofing mechanism to rescue PTC-containing transcripts

NMD is commonly thought of as an evolved mechanism to protect against ‘unwanted’ transcripts by recognizing that they contain premature stop codons. However, NMD might itself be the source of problems by reducing the dosage. Could NAS be an evolved quality control mechanism to prevent NMD operating on a particular subclass of genes? In some of the language concerning the scanning model in particular, a possible adaptive significance seems to be implicit. For example, Cartegni et al. (15) suggest that the process is there to verify the integrity of an ORF and, ‘when necessary, direct the splicing machinery to skip the offending exon’ (15). Wang et al (37) similarly refer to it as a ‘correction response’.

In many cases, the phenotypic consequences of splicing out an exon containing what would be a PTC should be less harmful than either degradation of the transcript or truncation of the protein, particularly if exon skipping maintains reading frame integrity. In this scenario, there could conceivably be selection for NAS. For example, nonsense mutations in the dystrophin-encoding *DMD* gene result in loss of functional protein (111) resulting in Duchenne muscular dystrophy (DMD). However, in Becker muscular dystrophy (BMD), which has a less severe phenotype (21,22,112–116), the PTC results in NAS encoding a shortened transcript but retaining the reading frame, restoring partial protein functionality. Similarly, the ability to express functional, yet shortened isoforms, such as *CEP290* exon-skipped isoforms, is correlated with disease severity (16–19).

However, NAS also affects exons that are not multiples of three long, and as we find, these often being pathogenic variants. Moreover, we find no evidence to suggest that PTCs associated with NAS in a ‘healthy’ context occur predominantly in exons of length 3n (see Supplementary Text S7). Given the relative rates of large-effect NAS, and that much of the variation in splicing associated with other PTCs is very small and likely a reflection of stochastic variation in exon inclusion, it seems unlikely that NAS is a genome-wide error-proofing mechanism under selection to rescue transcripts from NMD. Further, fitness benefits associated with the small variations in exon skipping PTCs are unlikely to be selectable. If PTC-containing transcripts derived from inherited mutations are particularly costly to fitness, the PTC-containing allele would likely be eliminated via purifying selection (although in rare and very specific cases variants are advantageous (117–119)). Thus, NAS is unlikely to be an evolutionarily conserved adaptive mechanism.

Our results are consistent with the alternative model, namely that NAS occurs as a consequence of ESE-binding proteins having to recognise a set of motifs that, due to being located within exons, by definition have a depletion of stop codons (40,67). Nonsense mutations thus break such interactions and cause unwanted splice disruption. However, this leaves unanswered the problem of why some nonsense mutations appear to be reading frame dependent in their ability to induce skipping (26). It could also be questioned why our prime candidates from 1000 Genomes data are not seen to hit ESEs more frequently. As we used a conservative set of ESEs it is possible that other motifs also function as splice enhancers but are not included in our set of motifs. Indeed, it could be that ‘weak’ motifs are associated with the weak NAS effects that we witness in the 1000 genome data, while ‘strong’ motifs cause more severe disruption and are thus associated with pathogenic effects. That the machine learning approaches find enrichment of splice defects in the 1000 genome NAS associated PTCs supports such a more nuanced model.

### The importance of accurate classification of nonsense mutations and their roles in therapeutics

Our results demonstrate the importance of understanding the broader implications for the classification of mutations. Even our conservative estimate suggests that the pathogenic effects of a significant proportion of nonsense mutations could be misunderstood. This data provides further evidence to suggest mutations in general, but SNPs in particular, should be routinely analysed at the mRNA level (15) prior to classification as mutations with seemingly no functional significance can be deleterious (120,121). This is particularly applicable to synonymous mutations, whose pathogenic significance might otherwise be overlooked - such mutations may disrupt ESEs or even create cryptic splice sites that result in a diseased phenotype (122–124) despite having no direct effect on the peptide sequence.

The consequences of correct classification of nonsense mutations might be best contextualised when considering therapeutic approaches to disease. A variety of therapies targeting nonsense mutations have been shown to restore protein function (125,126), however, these therapies are only effective if the PTC is present in the mature transcript. For example, a variety of diseases including Mucopolysaccharidosis type VI (MPS VI) (127), Usher syndrome (128,129) and DMD (130,131) are treated using strategies involving PTC124. This is thought to suppress translation termination at PTCs but not natural stop codons (132) and is therefore only effective if substantial levels of mRNA are available containing the PTC. However, if the PTC disrupts splicing and leads to exon skipping any such therapy is unlikely to be effective.

## DATA AVAILABILITY

All data used in the analyses are publicly available, with links to resources as described in the Material and Methods. Custom scripts used to perform analyses are available at http://github.com/rosinaSav/NAS_code.

## Supplementary Material

gkab750_Supplemental_Files

## References

[1] Jackson M., Marks L., May G.H.W., Wilson J.B. The genetic basis of disease. Essays Biochem. 2018; 62:643–723. 30509934 10.1042/EBC20170053 PMC6279436

[2] Price A.L., Spencer C.C.A., Donnelly P. Progress and promise in understanding the genetic basis of common diseases. Proc. R. Soc. B. 2015; 282:20151684. 10.1098/rspb.2015.1684 PMC4707742 26702037

[3] Ginsburg G.S., Phillips K.A. Precision medicine: from science to value. Health Aff. (Millwood). 2018; 37:694–701. 29733705 10.1377/hlthaff.2017.1624 PMC5989714

[4] Mort M., Ivanov D., Cooper D.N., Chuzhanova N.A. A meta-analysis of nonsense mutations causing human genetic disease. Hum. Mutat. 2008; 29:1037–1047. 18454449 10.1002/humu.20763

[5] Holbrook J.A., Neu-Yilik G., Hentze M.W., Kulozik A.E. Nonsense-mediated decay approaches the clinic. Nat. Genet. 2004; 36:801–808. 15284851 10.1038/ng1403

[6] Chung C.G., Lee H., Lee S.B. Mechanisms of protein toxicity in neurodegenerative diseases. Cell. Mol. Life Sci. 2018; 75:3159–3180. 29947927 10.1007/s00018-018-2854-4 PMC6063327

[7] Karam R., Carvalho J., Bruno I., Graziadio C., Senz J., Huntsman D., Carneiro F., Seruca R., Wilkinson M.F., Oliveira C. The NMD mRNA surveillance pathway downregulates aberrant E-cadherin transcripts in gastric cancer cells and in CDH1 mutation carriers. Oncogene. 2008; 27:4255–4260. 18427545 10.1038/onc.2008.62

[8] Maquat L.E. Nonsense-mediated mRNA decay in mammals. J. Cell Sci. 2005; 118:1773–1776. 15860725 10.1242/jcs.01701

[9] Brogna S., Wen J. Nonsense-mediated mRNA decay (NMD) mechanisms. Nat. Struct. Mol. Biol. 2009; 16:107–113. 19190664 10.1038/nsmb.1550

[10] Dietz H.C., Valle D., Francomano C.A., Kendzior R.J. Jr, Pyeritz R.E., Cutting G.R. The skipping of constitutive exons in vivo induced by nonsense mutations. Science. 1993; 259:680–683. 8430317 10.1126/science.8430317

[11] Valentine C.R. The association of nonsense codons with exon skipping. Mutat. Res. 1998; 411:87–117. 9806422 10.1016/s1383-5742(98)00010-6

[12] Maquat L.E. NASty effects on fibrillin pre-mRNA splicing: another case of ESE does it, but proposals for translation-dependent splice site choice live on. Genes Dev. 2002; 16:1743–1753. 12130534 10.1101/gad.1014502

[13] Hentze M.W., Kulozik A.E. A perfect message: RNA surveillance and nonsense-mediated decay. Cell. 1999; 96:307–310. 10025395 10.1016/s0092-8674(00)80542-5

[14] Anna A., Monika G. Splicing mutations in human genetic disorders: examples, detection, and confirmation. J. Appl. Genet. 2018; 59:253–268. 29680930 10.1007/s13353-018-0444-7 PMC6060985

[15] Cartegni L., Chew S.L., Krainer A.R. Listening to silence and understanding nonsense: exonic mutations that affect splicing. Nat. Rev. Genet. 2002; 3:285–298. 11967553 10.1038/nrg775

[16] Di Blasi C., He Y., Morandi L., Cornelio F., Guicheney P., Mora M. Mild muscular dystrophy due to a nonsense mutation in the LAMA2 gene resulting in exon skipping. Brain. 2001; 124:698–704. 11287370 10.1093/brain/124.4.698

[17] Littink K.W., Pott J.W., Collin R.W., Kroes H.Y., Verheij J.B., Blokland E.A., de Castro Miro M., Hoyng C.B., Klaver C.C., Koenekoop R.K. et al. A novel nonsense mutation in CEP290 induces exon skipping and leads to a relatively mild retinal phenotype. Invest. Ophthalmol. Vis. Sci. 2010; 51:3646–3652. 20130272 10.1167/iovs.09-5074

[18] Melis M.A., Muntoni F., Cau M., Loi D., Puddu A., Boccone L., Mateddu A., Cianchetti C., Cao A. Novel nonsense mutation (C→A nt 10512) in exon 72 of dystrophin gene leading to exon skipping in a patient with a mild dystrophinopathy. Hum. Mutat. 1998; 1998:S137–S138. 10.1002/humu.1380110146 9452067

[19] Pasmooij A.M., van Zalen S., Nijenhuis A.M., Kloosterhuis A.J., Zuiderveen J., Jonkman M.F., Pas H.H. A very mild form of non-Herlitz junctional epidermolysis bullosa: BP180 rescue by outsplicing of mutated exon 30 coding for the COL15 domain. Exp. Dermatol. 2004; 13:125–128. 15009107 10.1111/j.0906-6705.2004.00141.x

[20] Moseley C.T., Mullis P.E., Prince M.A., Phillips J.A. An Exon splice enhancer mutation causes autosomal dominant GH deficiency. J. Clin. Endocrinol. Metab. 2002; 87:847–852. 11836331 10.1210/jcem.87.2.8236

[21] Helderman-van den Enden A.T., Straathof C.S., Aartsma-Rus A., den Dunnen J.T., Verbist B.M., Bakker E., Verschuuren J.J., Ginjaar H.B. Becker muscular dystrophy patients with deletions around exon 51; a promising outlook for exon skipping therapy in Duchenne patients. Neuromuscul. Disord. 2010; 20:251–254. 20153965 10.1016/j.nmd.2010.01.013

[22] Shiga N., Takeshima Y., Sakamoto H., Inoue K., Yokota Y., Yokoyama M., Matsuo M. Disruption of the splicing enhancer sequence within exon 27 of the dystrophin gene by a nonsense mutation induces partial skipping of the exon and is responsible for Becker muscular dystrophy. J. Clin. Invest. 1997; 100:2204–2210. 9410897 10.1172/JCI119757 PMC508415

[23] Lorson C.L., Hahnen E., Androphy E.J., Wirth B. A single nucleotide in the SMN gene regulates splicing and is responsible for spinal muscular atrophy. Proc. Natl. Acad. Sci. U. S. A. 1999; 96:6307–6311. 10339583 10.1073/pnas.96.11.6307 PMC26877

[24] Xu W., Yang X., Hu X., Li S. Fifty-four novel mutations in the NF1 gene and integrated analyses of the mutations that modulate splicing. Int. J. Mol. Med. 2014; 34:53–60. 24789688 10.3892/ijmm.2014.1756 PMC4072343

[25] Urlaub G., Mitchell P.J., Ciudad C.J., Chasin L.A. Nonsense mutations in the dihydrofolate reductase gene affect RNA processing. Mol. Cell. Biol. 1989; 9:2868–2880. 2779551 10.1128/mcb.9.7.2868-2880.1989 PMC362753

[26] Dietz H.C., Kendzior R.J. Jr Maintenance of an open reading frame as an additional level of scrutiny during splice site selection. Nat. Genet. 1994; 8:183–188. 7842017 10.1038/ng1094-183

[27] Wilkinson M.F., Shyu A.B. RNA surveillance by nuclear scanning?. Nat. Cell Biol. 2002; 4:E144–E147. 12042827 10.1038/ncb0602-e144

[28] Apcher S., Millot G., Daskalogianni C., Scherl A., Manoury B., Fåhraeus R. Translation of pre-spliced RNAs in the nuclear compartment generates peptides for the MHC class I pathway. Proc. Natl. Acad. Sci. U.S.A. 2013; 110:17951. 24082107 10.1073/pnas.1309956110 PMC3816435

[29] David A., Dolan B.P., Hickman H.D., Knowlton J.J., Clavarino G., Pierre P., Bennink J.R., Yewdell J.W. Nuclear translation visualized by ribosome-bound nascent chain puromycylation. J. Cell Biol. 2012; 197:45–57. 22472439 10.1083/jcb.201112145 PMC3317795

[30] Al-Jubran K., Wen J., Abdullahi A., Roy Chaudhury S., Li M., Ramanathan P., Matina A., De S., Piechocki K., Rugjee K.N. et al. Visualization of the joining of ribosomal subunits reveals the presence of 80S ribosomes in the nucleus. RNA. 2013; 19:1669–1683. 24129492 10.1261/rna.038356.113 PMC3884666

[31] Iborra F.J., Jackson D.A., Cook P.R. Coupled transcription and translation within nuclei of mammalian cells. Science. 2001; 293:1139–1142. 11423616 10.1126/science.1061216

[32] Isken O., Maquat L.E. Quality control of eukaryotic mRNA: safeguarding cells from abnormal mRNA function. Genes Dev. 2007; 21:1833–3856. 17671086 10.1101/gad.1566807

[33] Shi M., Zhang H., Wang L.T., Zhu C.L., Sheng K., Du Y.H., Wang K., Dias A., Chen S., Whitman M. et al. Premature termination codons are recognized in the nucleus in a reading-frame-dependent manner. Cell Discov. 2015; 1:15001. 26491543 10.1038/celldisc.2015.1 PMC4610414

[34] Naeger L.K., Schoborg R.V., Zhao Q., Tullis G.E., Pintel D.J. Nonsense mutations inhibit splicing of MVM RNA in cis when they interrupt the reading frame of either exon of the final spliced product. Genes Dev. 1992; 6:1107–1119. 1592259 10.1101/gad.6.6.1107

[35] Aoufouchi S., Yelamos J., Milstein C. Nonsense mutations inhibit RNA splicing in a cell-free system: recognition of mutant codon is independent of protein synthesis. Cell. 1996; 85:415–422. 8616896 10.1016/s0092-8674(00)81119-8

[36] Carter M.S., Li S., Wilkinson M.F. A splicing-dependent regulatory mechanism that detects translation signals. EMBO J. 1996; 15:5965–5975. 8918474 PMC452383

[37] Wang J., Chang Y.F., Hamilton J.I., Wilkinson M.F. Nonsense-associated altered splicing: a frame-dependent response distinct from nonsense-mediated decay. Mol. Cell. 2002; 10:951–957. 12419238 10.1016/s1097-2765(02)00635-4

[38] Mohn F., Buhler M., Muhlemann O. Nonsense-associated alternative splicing of T-cell receptor beta genes: no evidence for frame dependence. RNA. 2005; 11:147–156. 15613535 10.1261/rna.7182905 PMC1370704

[39] Buhler M., Muhlemann O. Alternative splicing induced by nonsense mutations in the immunoglobulin mu VDJ exon is independent of truncation of the open reading frame. RNA. 2005; 11:139–146. 15613538 10.1261/rna.7183805 PMC1370703

[40] Abrahams L., Hurst L.D. A depletion of stop codons in lincRNA is owing to transfer of selective constraint from coding sequences. Mol. Biol. Evol. 2019; 37:1148–1164. 10.1093/molbev/msz299 PMC7086181 31841162

[41] Blencowe B.J. Exonic splicing enhancers: mechanism of action, diversity and role in human genetic diseases. Trends Biochem. Sci. 2000; 25:106–110. 10694877 10.1016/s0968-0004(00)01549-8

[42] Liu H.X., Cartegni L., Zhang M.Q., Krainer A.R. A mechanism for exon skipping caused by nonsense or missense mutations in BRCA1 and other genes. Nat. Genet. 2001; 27:55–58. 11137998 10.1038/83762

[43] Caputi M., Kendzior R.J. Jr, Beemon K.L. A nonsense mutation in the fibrillin-1 gene of a Marfan syndrome patient induces NMD and disrupts an exonic splicing enhancer. Genes Dev. 2002; 16:1754–1759. 12130535 10.1101/gad.997502 PMC186389

[44] Aznarez I., Zielenski J., Rommens J.M., Blencowe B.J., Tsui L.C. Exon skipping through the creation of a putative exonic splicing silencer as a consequence of the cystic fibrosis mutation R553X. J. Med. Genet. 2007; 44:341–346. 17475917 10.1136/jmg.2006.045880 PMC2597982

[45] Pagani F., Buratti E., Stuani C., Baralle F.E. Missense, nonsense, and neutral mutations define juxtaposed regulatory elements of splicing in cystic fibrosis transmembrane regulator exon 9. J. Biol. Chem. 2003; 278:26580–26588. 12732620 10.1074/jbc.M212813200

[46] Peterlongo P., Catucci I., Colombo M., Caleca L., Mucaki E., Bogliolo M., Marin M., Damiola F., Bernard L., Pensotti V. et al. FANCM c.5791C>T nonsense mutation (rs144567652) induces exon skipping, affects DNA repair activity and is a familial breast cancer risk factor. Hum. Mol. Genet. 2015; 24:5345–5355. 26130695 10.1093/hmg/ddv251 PMC4550823

[47] Zatkova A., Messiaen L., Vandenbroucke I., Wieser R., Fonatsch C., Krainer A.R., Wimmer K. Disruption of exonic splicing enhancer elements is the principal cause of exon skipping associated with seven nonsense or missense alleles of NF1. Hum. Mutat. 2004; 24:491–501. 15523642 10.1002/humu.20103

[48] Fairbrother W.G., Holste D., Burge C.B., Sharp P.A. Single nucleotide polymorphism-based validation of exonic splicing enhancers. PLoS Biol. 2004; 2:E268. 15340491 10.1371/journal.pbio.0020268 PMC514884

[49] Woolfe A., Mullikin J.C., Elnitski L. Genomic features defining exonic variants that modulate splicing. Genome Biol. 2010; 11:R20. 20158892 10.1186/gb-2010-11-2-r20 PMC2872880

[50] Rong S., Buerer L., Rhine C.L., Wang J., Cygan K.J., Fairbrother W.G. Mutational bias and the protein code shape the evolution of splicing enhancers. Nat. Commun. 2020; 11:2845. 32504065 10.1038/s41467-020-16673-z PMC7275064

[51] Fairbrother W.G., Yeh R.F., Sharp P.A., Burge C.B. Predictive identification of exonic splicing enhancers in human genes. Science. 2002; 297:1007–1013. 12114529 10.1126/science.1073774

[52] Caceres E.F., Hurst L.D. The evolution, impact and properties of exonic splice enhancers. Genome Biol. 2013; 14:R143. 24359918 10.1186/gb-2013-14-12-r143 PMC4054783

[53] Carlini D.B., Genut J.E. Synonymous SNPs provide evidence for selective constraint on human exonic splicing enhancers. J. Mol. Evol. 2006; 62:89–98. 16320116 10.1007/s00239-005-0055-x

[54] Parmley J.L., Chamary J.V., Hurst L.D. Evidence for purifying selection against synonymous mutations in mammalian exonic splicing enhancers. Mol. Biol. Evol. 2006; 23:301–309. 16221894 10.1093/molbev/msj035

[55] Parmley J.L., Urrutia A.O., Potrzebowski L., Kaessmann H., Hurst L.D. Splicing and the evolution of proteins in mammals. PLoS Biol. 2007; 5:e14. 17298171 10.1371/journal.pbio.0050014 PMC1790955

[56] Savisaar R., Hurst L.D. Exonic splice regulation imposes strong selection at synonymous sites. Genome Res. 2018; 28:1442–1454. 30143596 10.1101/gr.233999.117 PMC6169883

[57] Schüler A., Ghanbarian A.T., Hurst L.D. Purifying selection on splice-related motifs, not expression level nor RNA folding, explains nearly all constraint on human lincRNAs. Mol. Biol. Evol. 2014; 31:3164–3183. 25158797 10.1093/molbev/msu249 PMC4245815

[58] Savisaar R., Hurst L.D. Estimating the prevalence of functional exonic splice regulatory information. Hum. Genet. 2017; 136:1059–1078. 28405812 10.1007/s00439-017-1798-3 PMC5602102

[59] Supek F., Minana B., Valcarcel J., Gabaldon T., Lehner B. Synonymous mutations frequently act as driver mutations in human cancers. Cell. 2014; 156:1324–1335. 24630730 10.1016/j.cell.2014.01.051

[60] Sterne-Weiler T., Howard J., Mort M., Cooper D.N., Sanford J.R. Loss of exon identity is a common mechanism of human inherited disease. Genome Res. 2011; 21:1563–1571. 21750108 10.1101/gr.118638.110 PMC3202274

[61] Collin R.W.J., de Heer A.M.R., Oostrik J., Pauw R.J., Plantinga R.F., Huygen P.L., Admiraal R., de Brouwer A.P.M., Strom T.M., Cremers C. et al. Mid-frequency DFNA8/12 hearing loss caused by a synonymous TECTA mutation that affects an exonic splice enhancer. Eur. J. Hum. Genet. 2008; 16:1430–1436. 18575463 10.1038/ejhg.2008.110

[62] Lim K.H., Ferraris L., Filloux M.E., Raphael B.J., Fairbrother W.G. Using positional distribution to identify splicing elements and predict pre-mRNA processing defects in human genes. Proc. Natl. Acad. Sci. U.S.A. 2011; 108:11093–11098. 21685335 10.1073/pnas.1101135108 PMC3131313

[63] Ramser J., Abidi F.E., Burckle C.A., Lenski C., Toriello H., Wen G.P., Lubs H.A., Engert S., Stevenson R.E., Meindl A. et al. A unique exonic splice enhancer mutation in a family with X-linked mental retardation and epilepsy points to a novel role of the renin receptor. Hum. Mol. Genet. 2005; 14:1019–1027. 15746149 10.1093/hmg/ddi094

[64] Buchner D.A., Trudeau M., Meisler M.H. SCNM1, a putative RNA splicing factor that modifies disease severity in mice. Science. 2003; 301:967–969. 12920299 10.1126/science.1086187

[65] The 1000 Genomes Project Consortium A global reference for human genetic variation. Nature. 2015; 526:68–74. 26432245 10.1038/nature15393 PMC4750478

[66] Sultan M., Schulz M.H., Richard H., Magen A., Klingenhoff A., Scherf M., Seifert M., Borodina T., Soldatov A., Parkhomchuk D. et al. A global view of gene activity and alternative splicing by deep sequencing of the human transcriptome. Science. 2008; 321:956–960. 18599741 10.1126/science.1160342

[67] Soemedi R., Cygan K.J., Rhine C.L., Wang J., Bulacan C., Yang J., Bayrak-Toydemir P., McDonald J., Fairbrother W.G. Pathogenic variants that alter protein code often disrupt splicing. Nat. Genet. 2017; 49:848–855. 28416821 10.1038/ng.3837 PMC6679692

[68] Mou H., Smith J.L., Peng L., Yin H., Moore J., Zhang X.-O., Song C.-Q., Sheel A., Wu Q., Ozata D.M. et al. CRISPR/Cas9-mediated genome editing induces exon skipping by alternative splicing or exon deletion. Genome Biol. 2017; 18:108. 28615073 10.1186/s13059-017-1237-8 PMC5470253

[69] Sharpe J.J., Cooper T.A. Unexpected consequences: exon skipping caused by CRISPR-generated mutations. Genome Biol. 2017; 18:109. 28615035 10.1186/s13059-017-1240-0 PMC5470267

[70] Landrum M.J., Lee J.M., Benson M., Brown G.R., Chao C., Chitipiralla S., Gu B., Hart J., Hoffman D., Jang W. et al. ClinVar: improving access to variant interpretations and supporting evidence. Nucleic Acids Res. 2018; 46:D1062–D1067. 29165669 10.1093/nar/gkx1153 PMC5753237

[71] Wu X., Hurst L.D. Determinants of the usage of splice-associated cis-motifs predict the distribution of human pathogenic SNPs. Mol. Biol. Evol. 2016; 33:518–529. 26545919 10.1093/molbev/msv251 PMC4866546

[72] Cheng J., Nguyen T.Y.D., Cygan K.J., Celik M.H., Fairbrother W.G., Avsec Z., Gagneur J. MMSplice: modular modeling improves the predictions of genetic variant effects on splicing. Genome Biol. 2019; 20:48. 30823901 10.1186/s13059-019-1653-z PMC6396468

[73] Zerbino D.R., Achuthan P., Akanni W., Amode M.R., Barrell D., Bhai J., Billis K., Cummins C., Gall A., Giron C.G. et al. Ensembl 2018. Nucleic Acids Res. 2018; 46:D754–D761. 29155950 10.1093/nar/gkx1098 PMC5753206

[74] Lappalainen T., Sammeth M., Friedlander M.R., t Hoen P.A., Monlong J., Rivas M.A., Gonzalez-Porta M., Kurbatova N., Griebel T., Ferreira P.G. et al. Transcriptome and genome sequencing uncovers functional variation in humans. Nature. 2013; 501:506–511. 24037378 10.1038/nature12531 PMC3918453

[75] Kinsella R.J., Kähäri A., Haider S., Zamora J., Proctor G., Spudich G., Almeida-King J., Staines D., Derwent P., Kerhornou A. et al. Ensembl BioMarts: a hub for data retrieval across taxonomic space. Database (Oxford). 2011; 2011:bar030. 21785142 10.1093/database/bar030 PMC3170168

[76] van der Walt S., Colbert S.C., Varoquaux G. The NumPy array: a structure for efficient numerical computation. Comput. Sci. Eng. 2011; 13:22–30.

[77] R Core Team 2017; Vienna, Austria 4.3.2 ed. R Foundation for Statistical Computing.

[78] Quinlan A.R., Hall I.M. BEDTools: a flexible suite of utilities for comparing genomic features. Bioinformatics. 2010; 26:841–842. 20110278 10.1093/bioinformatics/btq033 PMC2832824

[79] Li H., Handsaker B., Wysoker A., Fennell T., Ruan J., Homer N., Marth G., Abecasis G., Durbin R. The sequence alignment/map format and SAMtools. Bioinformatics. 2009; 25:2078–2079. 19505943 10.1093/bioinformatics/btp352 PMC2723002

[80] Danecek P., Auton A., Abecasis G., Albers C.A., Banks E., DePristo M.A., Handsaker R.E., Lunter G., Marth G.T., Sherry S.T. et al. The variant call format and VCFtools. Bioinformatics. 2011; 27:2156–2158. 21653522 10.1093/bioinformatics/btr330 PMC3137218

[81] Li H. Tabix: fast retrieval of sequence features from generic TAB-delimited files. Bioinformatics. 2011; 27:718–719. 21208982 10.1093/bioinformatics/btq671 PMC3042176

[82] Dobin A., Davis C.A., Schlesinger F., Drenkow J., Zaleski C., Jha S., Batut P., Chaisson M., Gingeras T.R. STAR: ultrafast universal RNA-seq aligner. Bioinformatics. 2013; 29:15–21. 23104886 10.1093/bioinformatics/bts635 PMC3530905

[83] Mordstein C., Savisaar R., Young R.S., Bazile J., Talmane L., Luft J., Liss M., Taylor M.S., Hurst L.D., Kudla G. Codon usage and splicing jointly influence mRNA localization. Cell Syst. 2020; 10:351–362. 32275854 10.1016/j.cels.2020.03.001 PMC7181179

[84] Wang J., Gudikote J.P., Olivas O.R., Wilkinson M.F. Boundary-independent polar nonsense-mediated decay. EMBO Rep. 2002; 3:274–279. 11850396 10.1093/embo-reports/kvf036 PMC1084009

[85] Livak K.J., Schmittgen T.D. Analysis of relative gene expression data using real-time quantitative PCR and the 2−ΔΔCT method. Methods. 2001; 25:402–408. 11846609 10.1006/meth.2001.1262

[86] Landrum M.J., Lee J.M., Benson M., Brown G., Chao C., Chitipiralla S., Gu B., Hart J., Hoffman D., Hoover J. et al. ClinVar: public archive of interpretations of clinically relevant variants. Nucleic. Acids. Res. 2016; 44:D862–D868. 26582918 10.1093/nar/gkv1222 PMC4702865

[87] The Fantom Consortium, the Riken PMI, CLST Forrest A.R.R., Kawaji H., Rehli M., Kenneth Baillie J., de Hoon M.J.L., Haberle V., Lassmann T. et al. A promoter-level mammalian expression atlas. Nature. 2014; 507:462–470. 24670764 10.1038/nature13182 PMC4529748

[88] Shen S., Park J.W., Lu Z.X., Lin L., Henry M.D., Wu Y.N., Zhou Q., Xing Y. rMATS: robust and flexible detection of differential alternative splicing from replicate RNA-Seq data. Proc. Natl. Acad. Sci. U.S.A. 2014; 111:E5593–E5601. 25480548 10.1073/pnas.1419161111 PMC4280593

[89] Rosenberg A.B., Patwardhan R.P., Shendure J., Seelig G. Learning the sequence determinants of alternative splicing from millions of random sequences. Cell. 2015; 163:698–711. 26496609 10.1016/j.cell.2015.09.054

[90] Xiong H.Y., Alipanahi B., Lee L.J., Bretschneider H., Merico D., Yuen R.K.C., Hua Y., Gueroussov S., Najafabadi H.S., Hughes T.R. et al. The human splicing code reveals new insights into the genetic determinants of disease. Science. 2015; 347:1254806. 25525159 10.1126/science.1254806 PMC4362528

[91] Yeo G., Burge C.B. Maximum entropy modeling of short sequence motifs with applications to RNA splicing signals. J. Comput. Biol. 2004; 11:377–394. 15285897 10.1089/1066527041410418

[92] Parmley J.L., Hurst L.D. Exonic splicing regulatory elements skew synonymous codon usage near intron-exon boundaries in mammals. Mol. Biol. Evol. 2007; 24:1600–1603. 17525472 10.1093/molbev/msm104

[93] Graveley B.R., Hertel K.J., Maniatis T. A systematic analysis of the factors that determine the strength of pre-mRNA splicing enhancers. EMBO J. 1998; 17:6747–6756. 9822617 10.1093/emboj/17.22.6747 PMC1171020

[94] Nagy E., Maquat L.E. A rule for termination-codon position within intron-containing genes: when nonsense affects RNA abundance. Trends Biochem. Sci. 1998; 23:198–199. 9644970 10.1016/s0968-0004(98)01208-0

[95] Ke S., Shang S., Kalachikov S.M., Morozova I., Yu L., Russo J.J., Ju J., Chasin L.A. Quantitative evaluation of all hexamers as exonic splicing elements. Genome Res. 2011; 21:1360–1374. 21659425 10.1101/gr.119628.110 PMC3149502

[96] Krawczak M., Reiss J., Cooper D.N. The mutational spectrum of single base-pair substitutions in mRNA splice junctions of human genes: causes and consequences. Hum. Genet. 1992; 90:41–54. 1427786 10.1007/BF00210743

[97] Baralle D., Lucassen A., Buratti E. Missed threads. The impact of pre-mRNA splicing defects on clinical practice. EMBO Rep. 2009; 10:810–816. 19648957 10.1038/embor.2009.170 PMC2726684

[98] Beck S., Berner A.M., Bignell G., Bond M., Callanan M.J., Chervova O., Conde L., Corpas M., Ecker S., Elliott H.R. et al. Personal Genome Project UK (PGP-UK): a research and citizen science hybrid project in support of personalized medicine. BMC Med. Genet. 2018; 11:108. 10.1186/s12920-018-0423-1 PMC6257975 30482208

[99] Polakis P. Wnt signaling and cancer. Genes Dev. 2000; 14:1837–1851. 10921899

[100] Polakis P. Wnt signaling in cancer. Cold Spring Harb. Perspect. Biol. 2012; 4:a008052. 22438566 10.1101/cshperspect.a008052 PMC3331705

[101] Zhan T., Rindtorff N., Boutros M. Wnt signaling in cancer. Oncogene. 2016; 36:1461–1473. 27617575 10.1038/onc.2016.304 PMC5357762

[102] Reya T., Clevers H. Wnt signalling in stem cells and cancer. Nature. 2005; 434:843–850. 15829953 10.1038/nature03319

[103] Klaus A., Birchmeier W. Wnt signalling and its impact on development and cancer. Nat. Rev. Cancer. 2008; 8:387–398. 18432252 10.1038/nrc2389

[104] Taipale J., Beachy P.A. The Hedgehog and Wnt signalling pathways in cancer. Nature. 2001; 411:349–354. 11357142 10.1038/35077219

[105] Zhang X., Abreu J.G., Yokota C., MacDonald B.T., Singh S., Coburn K.L.A., Cheong S.-M., Zhang M.M., Ye Q.-Z., Hang H.C. et al. Tiki1 is required for head formation via Wnt cleavage-oxidation and inactivation. Cell. 2012; 149:1565–1577. 22726442 10.1016/j.cell.2012.04.039 PMC3383638

[106] Hattori N., Yoshino H., Tanaka M., Suzuki H., Mizuno Y. Genotype in the 24-kDa subunit gene (NDUFV2) of mitochondrial complex I and susceptibility to Parkinson disease. Genomics. 1998; 49:52–58. 9570948 10.1006/geno.1997.5192

[107] Cameron J.M., MacKay N., Feigenbaum A., Tarnopolsky M., Blaser S., Robinson B.H., Schulze A. Exome sequencing identifies complex I NDUFV2 mutations as a novel cause of Leigh syndrome. Eur. J. Paediatr. Neurol. 2015; 19:525–532. 26008862 10.1016/j.ejpn.2015.05.002

[108] Stanford S.M., Aleshin A.E., Zhang V., Ardecky R.J., Hedrick M.P., Zou J., Ganji S.R., Bliss M.R., Yamamoto F., Bobkov A.A. et al. Diabetes reversal by inhibition of the low-molecular-weight tyrosine phosphatase. Nat. Chem. Biol. 2017; 13:624–632. 28346406 10.1038/nchembio.2344 PMC5435566

[109] Gloria-Bottini F., Gerlini G., Lucarini N., Borgiani P., Amante A., La Torre M., Antonacci E., Bottini E. Phosphotyrosine protein phosphatases and diabetic pregnancy: an association between low molecular weight acid phosphatase and degree of glycemic control. Experientia. 1996; 52:340–343. 8620937 10.1007/BF01919537

[110] Wang Z., Rolish M.E., Yeo G., Tung V., Mawson M., Burge C.B. Systematic identification and analysis of exonic splicing silencers. Cell. 2004; 119:831–845. 15607979 10.1016/j.cell.2004.11.010

[111] Aartsma-Rus A., Ginjaar I.B., Bushby K. The importance of genetic diagnosis for Duchenne muscular dystrophy. J. Med. Genet. 2016; 53:145–151. 26754139 10.1136/jmedgenet-2015-103387 PMC4789806

[112] Flanigan K.M., Dunn D.M., von Niederhausern A., Soltanzadeh P., Howard M.T., Sampson J.B., Swoboda K.J., Bromberg M.B., Mendell J.R., Taylor L.E. et al. Nonsense mutation-associated Becker muscular dystrophy: interplay between exon definition and splicing regulatory elements within the DMD gene. Hum. Mutat. 2011; 32:299–308. 21972111 10.1002/humu.21426 PMC3724403

[113] Moore R.S., Tirupathi S., Herron B., Sands A., Morrison P.J. Dystrophin exon 29 nonsense mutations cause a variably mild phenotype. Ulster Med. J. 2017; 86:185–188. 29581631 PMC5849976

[114] Carsana A., Frisso G., Tremolaterra M.R., Lanzillo R., Vitale D.F., Santoro L., Salvatore F. Analysis of dystrophin gene deletions indicates that the hinge III region of the protein correlates with disease severity. Ann. Hum. Genet. 2005; 69:253–259. 15845029 10.1046/J.1469-1809.2005.00160.x

[115] Anthony K., Arechavala-Gomeza V., Ricotti V., Torelli S., Feng L., Janghra N., Tasca G., Guglieri M., Barresi R., Armaroli A. et al. Biochemical characterization of patients with in-frame or out-of-frame DMD deletions pertinent to exon 44 or 45 skipping. JAMA Neurol. 2014; 71:32–40. 24217213 10.1001/jamaneurol.2013.4908

[116] Bello L., Campadello P., Barp A., Fanin M., Semplicini C., Soraru G., Caumo L., Calore C., Angelini C., Pegoraro E. Functional changes in Becker muscular dystrophy: implications for clinical trials in dystrophinopathies. Sci. Rep. 2016; 6:32439. 27582364 10.1038/srep32439 PMC5007528

[117] North K.N., Yang N., Wattanasirichaigoon D., Mills M., Easteal S., Beggs A.H. A common nonsense mutation results in α-actinin-3 deficiency in the general population. Nat. Genet. 1999; 21:353–354. 10192379 10.1038/7675

[118] Yang N., MacArthur D.G., Gulbin J.P., Hahn A.G., Beggs A.H., Easteal S., North K. ACTN3 genotype is associated with human elite athletic performance. Am. J. Hum. Genet. 2003; 73:627–631. 12879365 10.1086/377590 PMC1180686

[119] Hawn T.R., Wu H., Grossman J.M., Hahn B.H., Tsao B.P., Aderem A. A stop codon polymorphism of Toll-like receptor 5 is associated with resistance to systemic lupus erythematosus. Proc. Natl. Acad. Sci. U.S A. 2005; 102:10593–10597. 16027372 10.1073/pnas.0501165102 PMC1180760

[120] Pfarr N., Prawitt D., Kirschfink M., Schroff C., Knuf M., Habermehl P., Mannhardt W., Zepp F., Fairbrother W.G., Loos M. et al. Linking C5 deficiency to an exonic splicing enhancer mutation. J. Immunol. 2005; 174:4172–4177. 15778377 10.4049/jimmunol.174.7.4172

[121] Fackenthal J.D., Cartegni L., Krainer A.R., Olopade O.I. BRCA2 T2722R is a deleterious allele that causes exon skipping. Am. J. Hum. Genet. 2002; 71:625–631. 12145750 10.1086/342192 PMC379197

[122] Austin F., Oyarbide U., Massey G., Grimes M., Corey S.J. Synonymous mutation in TP53 results in a cryptic splice site affecting its DNA-binding site in an adolescent with two primary sarcomas. Pediatr. Blood Cancer. 2017; 64:e26584. 10.1002/pbc.26584 PMC5937697 28475293

[123] Rice G.I., Reijns M.A., Coffin S.R., Forte G.M., Anderson B.H., Szynkiewicz M., Gornall H., Gent D., Leitch A., Botella M.P. et al. Synonymous mutations in RNASEH2A create cryptic splice sites impairing RNase H2 enzyme function in Aicardi-Goutieres syndrome. Hum. Mutat. 2013; 34:1066–1070. 23592335 10.1002/humu.22336 PMC3714325

[124] Sheikh T.I., Mittal K., Willis M.J., Vincent J.B. A synonymous change, p.Gly16Gly in MECP2 Exon 1, causes a cryptic splice event in a Rett syndrome patient. Orphanet J. Rare Dis. 2013; 8:108. 23866855 10.1186/1750-1172-8-108 PMC3729535

[125] Keeling K.M., Xue X., Gunn G., Bedwell D.M. Therapeutics based on stop codon readthrough. Annu. Rev. Genomics Hum. Genet. 2014; 15:371–394. 24773318 10.1146/annurev-genom-091212-153527 PMC5304456

[126] Dabrowski M., Bukowy-Bieryllo Z., Zietkiewicz E. Advances in therapeutic use of a drug-stimulated translational readthrough of premature termination codons. Mol. Med. 2018; 24:25. 30134808 10.1186/s10020-018-0024-7 PMC6016875

[127] Bartolomeo R., Polishchuk E.V., Volpi N., Polishchuk R.S., Auricchio A. Pharmacological read-through of nonsense ARSB mutations as a potential therapeutic approach for mucopolysaccharidosis VI. J. Inher. Metab. Dis. 2013; 36:363–371. 22971959 10.1007/s10545-012-9521-y PMC3590409

[128] Goldmann T., Overlack N., Möller F., Belakhov V., van Wyk M., Baasov T., Wolfrum U., Nagel-Wolfrum K. A comparative evaluation of NB30, NB54 and PTC124 in translational read-through efficacy for treatment of an USH1C nonsense mutation. EMBO Mol. Med. 2012; 4:1186–1199. 23027640 10.1002/emmm.201201438 PMC3494875

[129] Goldmann T., Overlack N., Wolfrum U., Nagel-Wolfrum K. PTC124-mediated translational readthrough of a nonsense mutation causing Usher syndrome type 1C. Hum. Gene Ther. 2011; 22:537–547. 21235327 10.1089/hum.2010.067

[130] Yukihara M., Ito K., Tanoue O., Goto K., Matsushita T., Matsumoto Y., Masuda M., Kimura S., Ueoka R. Effective drug delivery system for duchenne muscular dystrophy using hybrid liposomes including gentamicin along with reduced toxicity. Biol. Pharm. Bull. 2011; 34:712–716. 21532162 10.1248/bpb.34.712

[131] Finkel R.S., Flanigan K.M., Wong B., Bonnemann C., Sampson J., Sweeney H.L., Reha A., Northcutt V.J., Elfring G., Barth J. et al. Phase 2a study of ataluren-mediated dystrophin production in patients with nonsense mutation Duchenne muscular dystrophy. PLoS One. 2013; 8:e81302. 24349052 10.1371/journal.pone.0081302 PMC3859499

[132] Welch E.M., Barton E.R., Zhuo J., Tomizawa Y., Friesen W.J., Trifillis P., Paushkin S., Patel M., Trotta C.R., Hwang S. et al. PTC124 targets genetic disorders caused by nonsense mutations. Nature. 2007; 447:87. 17450125 10.1038/nature05756

[133] Kouprina N., Pavlicek A., Collins N.K., Nakano M., Noskov V.N., Ohzeki J.-I., Mochida G.H., Risinger J.I., Goldsmith P., Gunsior M. et al. The microcephaly ASPM gene is expressed in proliferating tissues and encodes for a mitotic spindle protein. Hum. Mol. Genet. 2005; 14:2155–2165. 15972725 10.1093/hmg/ddi220

[134] Macia M.S., Halbritter J., Delous M., Bredrup C., Gutter A., Filhol E., Mellgren A.E.C., Leh S., Bizet A., Braun D.A. et al. Mutations in MAPKBP1 Cause Juvenile or Late-Onset Cilia-Independent Nephronophthisis. Am. J. Hum. Genet. 2017; 100:323–333. 28089251 10.1016/j.ajhg.2016.12.011 PMC5294754

[135] Helias V., Saison C., Ballif B.A., Peyrard T., Takahashi J., Takahashi H., Tanaka M., Deybach J.-C., Puy H., Le Gall M. et al. ABCB6 is dispensable for erythropoiesis and specifies the new blood group system Langereis. Nat. Genet. 2012; 44:170. 22246506 10.1038/ng.1069 PMC3664204

[136] Boswell-Casteel R.C., Fukuda Y., Schuetz J.D. ABCB6, an ABC transporter impacting drug response and disease. The AAPS Journal. 2017; 20:8. 29192381 10.1208/s12248-017-0165-6 PMC5821141

[137] Kelter G., Steinbach D., Konkimalla V.B., Tahara T., Taketani S., Fiebig H.H., Efferth T. Role of transferrin receptor and the ABC transporters ABCB6 and ABCB7 for resistance and differentiation of tumor cells towards artesunate. PLoS One. 2007; 2:e798. 17726528 10.1371/journal.pone.0000798 PMC1949049

[138] Golmard L., Caux-Moncoutier V., Davy G., Al Ageeli E., Poirot B., Tirapo C., Michaux D., Barbaroux C., Enghien C.D., Nicolas A. et al. Germline mutation in the RAD51B gene confers predisposition to breast cancer. BMC Cancer. 2013; 13:484. 24139550 10.1186/1471-2407-13-484 PMC4016303

[139] Fisher C.W., Fisher C.R., Chuang J.L., Lau K.S., Chuang D.T., Cox R.P. Occurrence of a 2-bp (AT) deletion allele and a nonsense (G-to-T) mutant allele at the E2 (DBT) locus of six patients with maple syrup urine disease: multiple-exon skipping as a secondary effect of the mutations. Am. J. Hum. Genet. 1993; 52:414–424. 8430702 PMC1682180

