# Supplementary material for: Evidence in disease and non-disease contexts that nonsense mutations cause altered splicing via motif disruption
Source: Nucleic Acids Res. 2021 Sep 1;49(17):9665–85. doi: 10.1093/nar/gkab750 (PMC8464065; doi:10.1093/nar/gkab750)
Supplement: gkab750_Supplemental_Files [file gkab750_supplemental_files.zip › Legends for other supplements copy.pdf]

Legends for non-pdf spreadsheets

Supplementary data 1. Details of the 541 exons bearing nonsense mutations

Supplementary data 2. The sequences of the 541 exons bearing nonsense mutations

Supplementary Spreadsheets:
Sheet 1: Frequencies of mutations generating stop codons
Sheet 2: Illustration of possible problem owing to NMD
Sheet 3: Exon positions of ClinVar variants
Sheet 4: Statistics on the distribution of clinvar nonsense mutations
Sheet 5: Sequence of the minigene constructs
Sheet 6: PCR Primers used
Sheet 7: Genes that contain PTCs tend to be more tissue specific than the genes containing pPTCs
