# Supplementary material for: Evidence in disease and non-disease contexts that nonsense mutations cause altered splicing via motif disruption
Source: Nucleic Acids Res. 2021 Sep 1;49(17):9665–85. doi: 10.1093/nar/gkab750 (PMC8464065; doi:10.1093/nar/gkab750)
Supplement: gkab750_Supplemental_Files [file gkab750_supplemental_files.zip › Supplement_17082021.pdf]

Supplementary Texts

Supplementary Text 1: Nucleotide biases in the mutations that create PTCs may account for changes in PSI

While PTCs with large effects on splicing are mostly consistent with NAS, this effect may be a result of the nucleotides involved in the mutations generating PTCs. Indeed, the relative proportions of the different mutation classes generating PTCs (e.g. A$\rightarrow$T, C$\rightarrow$T) are significantly different than for mutations creating synonymous and nonsynonymous variants in the same set of exons (N = 1,458 non-nonsense mutations, χ^2 = 481.192, P = 5.680 $\times$ 10^{-104}, chi-squared test, Supplementary Spreadsheet 1, Supplementary Spreadsheets). This is robust to excluding N$\rightarrow$C mutations (N = 216) that cannot generate a PTC (χ^2 = 329.757, P = 2.480 $\times$ 10^{-72}, chi-squared test, Supplementary Spreadsheet 1, Supplementary Spreadsheets). Thus, the previous result may not be specific to PTCs, but more generally to nucleotides involved in the mutations that generate PTCs. Furthermore, there exists a bias towards A nucleotides in the three stop codons (44.44%) and ESEs (46.27%), with the PTC generating mutations also exhibiting such a bias (33.13%, Supplementary Spreadsheet 1, Supplementary Spreadsheets).

Thus, although stop codons tend to be depleted in ESEs (1), it is possible that mutations introducing PTCs may sometimes create ESEs leading to increased exon inclusion. More generally, while ESEs have a low density of stop codons (and so typically a PTC should cause exon skipping), a few ESEs do have one stop codon thus rendering ESE creation a possible explanation of instances where exon inclusion is increased.

Supplementary Text 2: Increases in PSI could be attributable to NMD

By definition, the PSI metric is dependent on the number of reads for the full-length isoform. Yet, full-length transcripts containing a PTC are likely to be removed at a particular rate by NMD. In this scenario, it is possible for the relative proportion of reads demonstrating exon inclusion (PSI) to decrease, despite no increase in the absolute number of skipped reads and actual number of PTC associated skipping events (see Supplementary Spreadsheet 2, Supplementary Spreadsheets for an example).

Thus, although we might observe a relative change in the rate of exon skipping between variants, there might be no absolute change in the number of exons skipped. It is imperative that any method to establish the effect of NAS therefore accounts for any potential effects of NMD.

Supplementary Text 3: Defining the large-effect threshold

We find many differences in PSI (ΔPSI) small, with differences in the overall degree to which a particular exon is skipped unlikely to have any phenotypically meaningful impact. In order to filter cases where there is likely to be an effect, we need to apply a lower bound ΔPSI threshold for which cases with differences above the threshold are considered to be meaningful in terms of exon skipping. A 5% difference threshold was chosen. Note, the result of large effect cases being consistent with increased exon skipping if the PTC is present (by one-tailed exact binomial test) is robust to threshold choice until reaching a lower-limit threshold of $\approx$ 0.7% (Supplementary Figure 2). Further, the significance of the result at 5% lies close to the threshold with strongest significance and minimal P-values (5.5% - 6.2%).

Thus, despite the threshold being arbitrarily defined, a significant P-value in the direction consistent with NAS is not simply an artefact of limiting results to a high threshold generating a significant result. Second, this threshold eliminates smaller-effect cases that when included contribute to a significant result in the direction consistent with NAS. By setting the threshold at 5% as a lower bound, we also the include all cases where the effect is strongest at slightly higher thresholds (5.5% - 6.2%) which would be excluded if set even 1.5% higher. Therefore, although user-defined, the 5% threshold is therefore appropriate for identifying cases whereby the effects of the PTC on exon skipping are likely to have important effects. Finally, a 5% threshold has previously been used in alternative splicing studies (2).

To match the number of large-effect PSI cases (50), we define a large change in RPMskip as one of greater than 0.026 units in either direction.

Supplementary Text 4: Similar NAS trends are observed in an independent dataset

We attempted to replicate this result using an alternative data source (3). We applied our pipeline to the 58 samples from the E-GEOD-19480 dataset that could be processed, aligning the raw reads to the reference genome using the STAR aligner. In these samples, we identify 272 PTCs. However, after processing the variants we are only able to detect splicing in 79 variants, of which 66 (83.54%) have no change in PSI. Whilst this is higher than 44.18% of variants with no change in the main analysis, this second dataset suffers from a relative lack of read depth when compared with the equivalent data in the original analysis (median Geuvadis reads per bam = 56,559,352, median E-GEOD-19480 reads per bam = 11,730,294, P = 1.14×10^{-14}, paired Wilcoxon signed rank test). This renders this dataset very underpowered. However, the absolute median change in PSI for variants increasing skipping (N = 4, median ΔPSI = 29.89) is greater than for those in the opposite direction (N = 9, median ΔPSI = 3.43) and almost significant (P = 0.093, Wilcoxon rank sum test), despite the lack of power. Again, 66 variants have no change in ΔRPMskip, however the 4 variants consistent with exon skipping have a significantly greater absolute effect (median ΔRPMskip = 0.308) when compared with variants displaying the opposite effect (median ΔRPMskip = 0.020). Three of these variants displaying both ΔPSI and ΔRPMskip in the direction consistent with NAS greater than the large-effect thresholds we defined (3.79%). Thus, while we find that we can replicate the results (estimate 4%), we strongly caution drawing on the analysis due to the relative lack of power.

Supplementary Text 5: Pathogenic and likely pathogenic variants occur in exon flanks more frequently than expected by chance

If PTCs frequently disrupt splicing due to the disruption of ESEs, we would expect PTCs to occur more frequently in the flanks of exons where ESEs are typically located. Using the set of ClinVar variants (see Methods), we find 68.49% (4,352/6,354) pathogenic nonsense mutations are located in the exon flank nucleotides where ESEs are typically located (nucleotides 3-69), despite the flanks only accounting for 55.66% (422,017/758,223) of total coding nucleotides. This frequency is significantly more than expected when comparing locations of other SNPs (χ^2 = 578.140, P = 2.870 $\times$ 10^{-126}, chi-squared test, Supplementary Spreadsheet 3, Supplementary Spreadsheets). This flank effect is also true of the likely pathogenic mutations (787/1,075 (73.21%) occur in the 111,002/264,391 (41.98%) flanking nucleotides, χ^2 = 539.590, P = 6.750 $\times$ 10^{-118}, chi-squared test, Supplementary Spreadsheet 3, Supplementary Spreadsheets).

These results are robust to removing "short" exons (those with a length shorter than 138 nucleotides, so the remaining exons include splice sites and exon flanks as well as an exon core region), as short exons could be defined as all "exon flank" (Supplementary Spreadsheet 3, Supplementary Spreadsheets). A similar skew towards exon ends has been seen for missense mutations (4), while the opposite is observed for SNPs that disrupt ESEs circulating in the population (5-7), consistent with selection against mutations that disrupt splicing.

Although suggestive, this exon flank bias may be a result of a nucleotide-related mutational bias towards exon ends (8). To control for this, we performed 10,000 simulations in which every real variant was replaced with a randomly chosen nucleotide from the same exon that matched the reference allele of the nonsense mutation, ensuring that each matched nucleotide is not also a disease-associated SNP. For each randomised set of simulated mutations, we then asked where in exons they were located. The real number of pathogenic nonsense mutations in exon flanks is significantly higher when compared with the number expected from the simulations (Z = 2.217, P $\approx$ 0.013, one-tailed empirical P-value, Supplementary Figure 4). However, the effect is less pronounced and nonsignificant for the likely pathogenic variants (Z = 0.756, P $\approx$ 0.238, one-tailed empirical P-value). As expected, for the 1000 Genomes PTC mutations we find a significant depletion of mutations in exon flanks (Z = -2.039, P $\approx$ 0.019, one-tailed empirical P-value). Thus, nonsense mutations that occur in the exon flanks, and are therefore more prone to disrupt splicing, are typically pathogenic.

Supplementary Text 6: Pathogenic PTCs occur in non 3n exons more frequently when the exon is long, with exons disrupted by pathogenic PTCs typically having greater ESE density

We also hypothesize that NAS may be more detrimental if the skipped exon is not of length 3n, as in these cases exon skipping would disrupt the reading frame. We find the exons hit by pathogenic nonsense variants (N = 3,572; note some exons are hit by multiple variants) are of length 3n (1,440/3,572 = 40.31%) as frequently than the exons hit by pathogenic non-nonsense variants (1,960/4,783 = 40.98%) (χ^2 = 0.348, P = 0.556, chi-squared test). Further, exons containing at least one pathogenic nonsense variant predicted to hit an INT3 ESE are as likely to be of length 3n (375/905, 40.44%) as exons in which none of the pathogenic nonsense variants are predicted to hit an ESE (1,065/2,667, 39.93%) (χ^2 = 0.574, P = 0.449, chi-squared test). However, we do find that variants in exons longer than 138 bp are more likely to occur in the exon flanks when in non-3n exons (1,418/2,360 = 60.08%) than when in 3n exons (909/1,771 = 51.33%) (χ^2 = 31.191, P = 2.339×10^{-8}, chi-squared test). The latter result is consistent with pathogenicity being disproportionately associated with nonsense induced splice disruption in exons that, when skipped, cause a downstream frameshift.

Potentially in agreement with the above result, we also find that exons containing pathogenic nonsense variants have a significantly greater ESE density (number of nucleotides contributing to an ESE per bp of sequence) than for the non-nonsense variants (median pathogenic nonsense exon ESE density = 0.171, median pathogenic non-nonsense exon ESE density = 0.159, P = 2.716×10^{-8}, Wilcoxon rank sum test). For reasons unknown, exons affected by pathogenic nonsense mutations are longer than exons affected exclusively by pathogenic synonymous and nonsynonymous variants (P < 2.2×10^{-16}, Wilcoxon rank sum test).

Supplementary Text 7: NAS is not an adaptive mechanism to save transcripts from NMD, whilst exons susceptible to NAS appear to be unremarkable

If exon skipping is an adaptive mechanism to save transcripts from NMD, we might expect the large effect exons to be more frequently of length three the exons for the other PTCs. We find this not to be the case when sampling 30 random exons from the non-large-effect PTCs 10,000 times ($P \approx 0.186$, one-tailed empirical P-value). Equally, if adaptive, we might expect the large effect cases to occur in shorter exons so if skipped it would have a smaller impact on the resulting protein, but we find no significant difference when comparing the median length with median length in the simulation exon sets ($P \approx 0.662$, one-tailed empirical P-value).

Additional examples of possible exceptionalism all suggest these exons have no particular defining characteristic. For example, one would expect ESEs to be more frequently disrupted by the set of PTCs in the large effect cases than those with little difference between PTC-/- and PTC-/+ variants. Using the INT3 ESE set (5), we find 6/30 PTCs hit a motif that resembles an ESE, however this number of hits is not significantly more than expected when taking 10,000 random sets of 30 of the remaining PTCs and asking how many have an equal or greater number of ESE hits than the real hits ($P \approx 0.299$, one-tailed empirical P-value). This result, however, is dependent on ESEs being functional in these exons. An alternative approach is to look where in exons the PTCs are located. Are those in the prime set found more frequently in the ESE hotspot region? 23/30 are in the 3-69bp region, although this is not significantly different to the number found in the region when comparing with 10,000 randomly chosen sets of 30 of the remaining PTCs ($P \approx 0.425$, one-tailed empirical P-value). Neither do we find a bias for PTCs located at a particular end of the exon (15 and 15 at the 5' and 3' ends respectively), again not significantly different to 10,000 simulants ($P \approx 0.703$, one-tailed empirical P-value).

Thus, it appears that the exons in which we observe large increases in exon skipping when associated with the PTC are not exceptional. However, as a proportion of mutations are tolerated in genes that are either lowly expressed or are of less importance, it could be the case that these mutations simply occur in exons of lowly expressed genes or those in which there is little phenotypic consequence. Indeed, the genes that contain PTCs tend to be more tissue specific than the genes containing pPTCs (see Supplementary Spreadsheet 7, Supplementary Spreadsheets).

References

1. Abrahams L, Hurst LD. A Depletion of Stop Codons in lincRNA is Owing to Transfer of Selective Constraint from Coding Sequences. Mol Biol Evol. 2019;37(4):1148-64.
2. Shen S, Park JW, Lu Z-x, Lin L, Henry MD, Wu YN, et al. rMATS: Robust and flexible detection of differential alternative splicing from replicate RNA-Seq data. Proceedings of the National Academy of Sciences. 2014;111(51):E5593-E601.
3. Pickrell JK, Marioni JC, Pai AA, Degner JF, Engelhardt BE, Nkadori E, et al. Understanding mechanisms underlying human gene expression variation with RNA sequencing. Nature. 2010;464(7289):768-72.
4. Wu X, Hurst LD. Determinants of the Usage of Splice-Associated cis-Motifs Predict the Distribution of Human Pathogenic SNPs. Mol Biol Evol. 2016;33(2):518-29.
5. Caceres EF, Hurst LD. The evolution, impact and properties of exonic splice enhancers. Genome Biol. 2013;14(12):R143.
6. Fairbrother WG, Holste D, Burge CB, Sharp PA. Single nucleotide polymorphism-based validation of exonic splicing enhancers. PLoS Biol. 2004;2(9):E268.
7. Carlini DB, Genut JE. Synonymous SNPs provide evidence for selective constraint on human exonic splicing enhancers. J Mol Evol. 2006;62(1):89-98.
8. Chamary JV, Hurst LD. Biased codon usage near intron-exon junctions: selection on splicing enhancers, splice-site recognition or something else? Trends Genet. 2005;21(5):256-9

Supplementary Figures

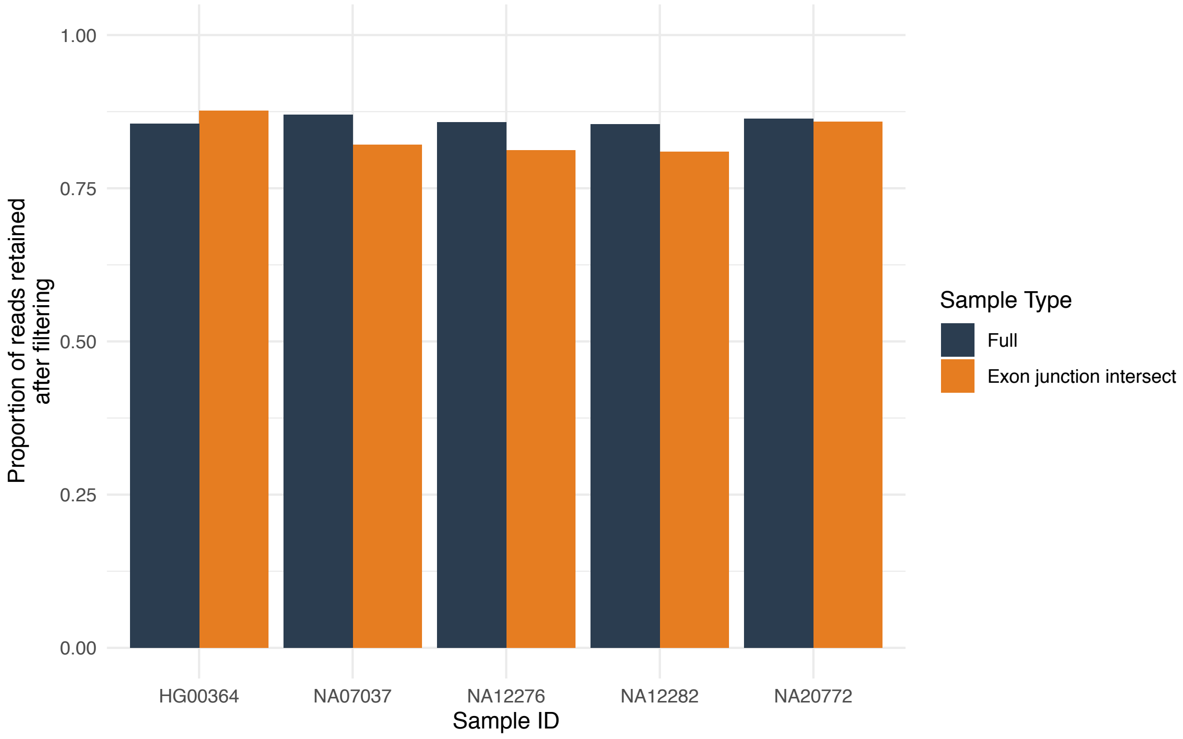

Supplementary Figure 1: Proportion of reads retained after BAM filtering for both full samples and samples filtered to retain only exon junction reads.

The proportion of reads retained following quality filtering of the BAM files for 5 randomly sampled files. All files retain similar levels of reads after each filtering with differences not significant (P = 0.188, paired Wilcoxon signed-rank test), suggesting the approach used to approximate the number of reads retained following filtering of the intersect file can be appropriately applied to estimate the number of reads retained after filtering of the whole file.

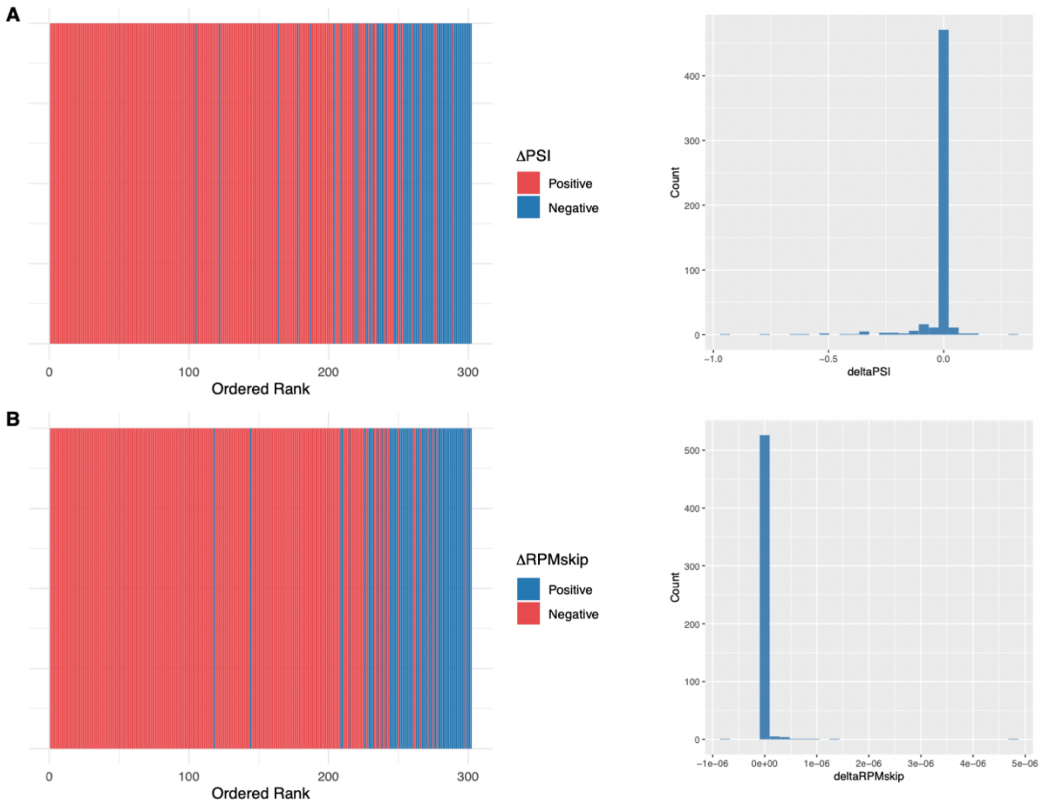

Supplementary Figure 2: Ordered rank scores and distributions for ΔPSI and ΔRPMskip. Rankings shown in blue are consistent with NAS and those shown in red in the opposite direction.

(A) Left panel: Ordered ranks of ascending absolute ΔPSI scores for exons with ΔPSI not equal to zero. Exons with increased exon skipping consistent with NAS are distributed towards the higher ordered ranks. However, due to the relatively few data points (N = 68) the absolute sum of ordered ranks (17,442) for ΔPSI < 0 scores is less than that for the larger group (N = 234) of ΔPSI > 0 scores (28,331). Right panel: histogram of same data but including zero values. (B) Left panel: Ordered ranks of ascending absolute ΔRPMskip scores for the exons where ΔRPMskip is not equal to zero. Exons with ΔRPMskip consistent with NAS also rank high, but again the sum of absolute ranks for positive ΔRPMskip scores (16,704) is less than the sum of absolute ranks for negative ΔRPMskip scores (29,049). Right panel: histogram of same data but including zero values.

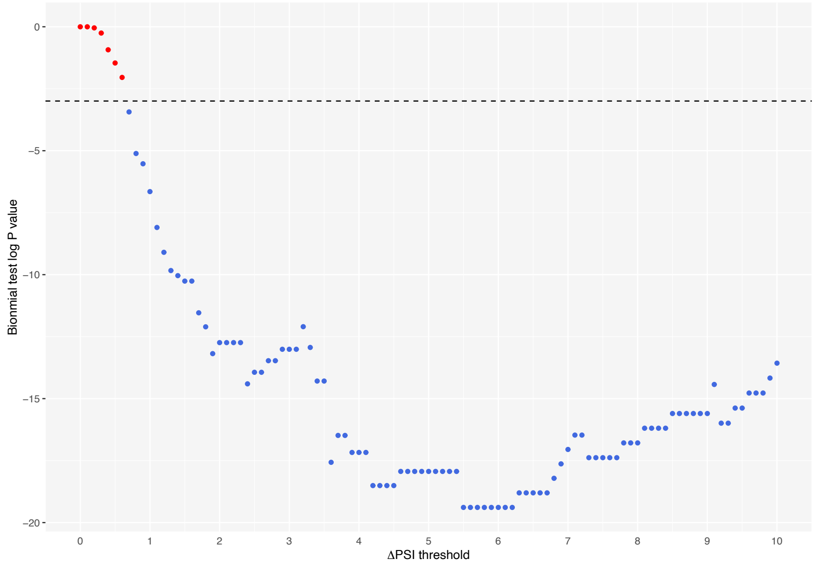

Supplementary Figure 3: Determining the large-effect ΔPSI threshold

P values for one-tailed exact binomial tests asking whether the number of PTCs for showing ΔPSI above increasing ΔPSI threshold in the direction consistent with NAS is significant. Thresholds above ≈0.7% demonstrate this effect.

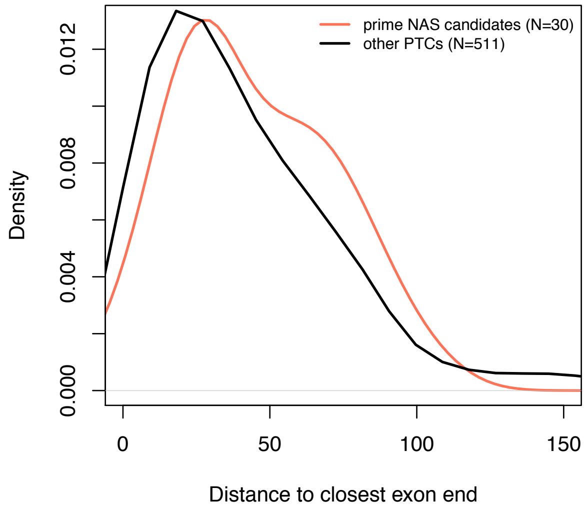

Supplementary Figure 4

Distance to the nearest exon end of the 30 prime NAS candidates and the 511 other PTCs. The difference between the two distributions is not statistically significant (P ~ 0.429 from a two-tailed Welch's t-test).

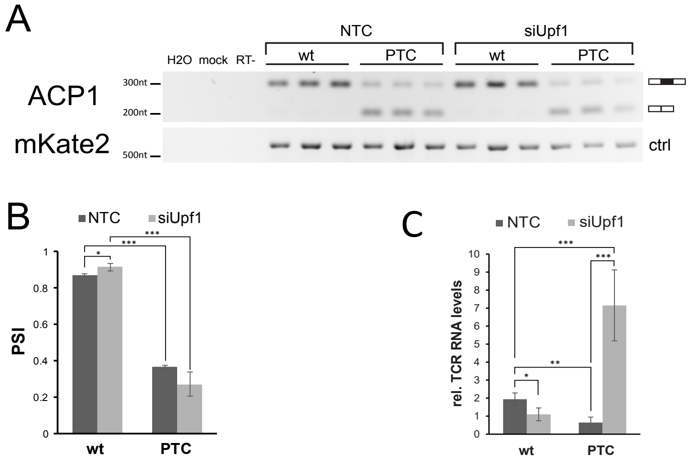

Supplementary Figure 5: Experimental expression of *ACP1* minigene constructs in Hek293T cells.

(A) Agarose gel electrophoresis of RT-PCR of Hek293T cells treated with either a non-targeting siRNA pool control (NTC) or Upf1-targeting siRNA (siUpf1). mKate2 levels are shown as transfection control. (B) PSI levels for wt and PTC-containing *ACP1* variants. Error bars denote the standard error of the mean. (C) qRT-PCR analysis of TCR-beta NMD-reporter constructs. RNA levels of TCR-beta are normalised to Gapdh. Error bars denote the standard error of the mean.

Supplementary Figure 6: Full gel images of those presented in in text Fig 4B. A) ACP1 Long exposure B) ACP1 normal exposure C) mKate2

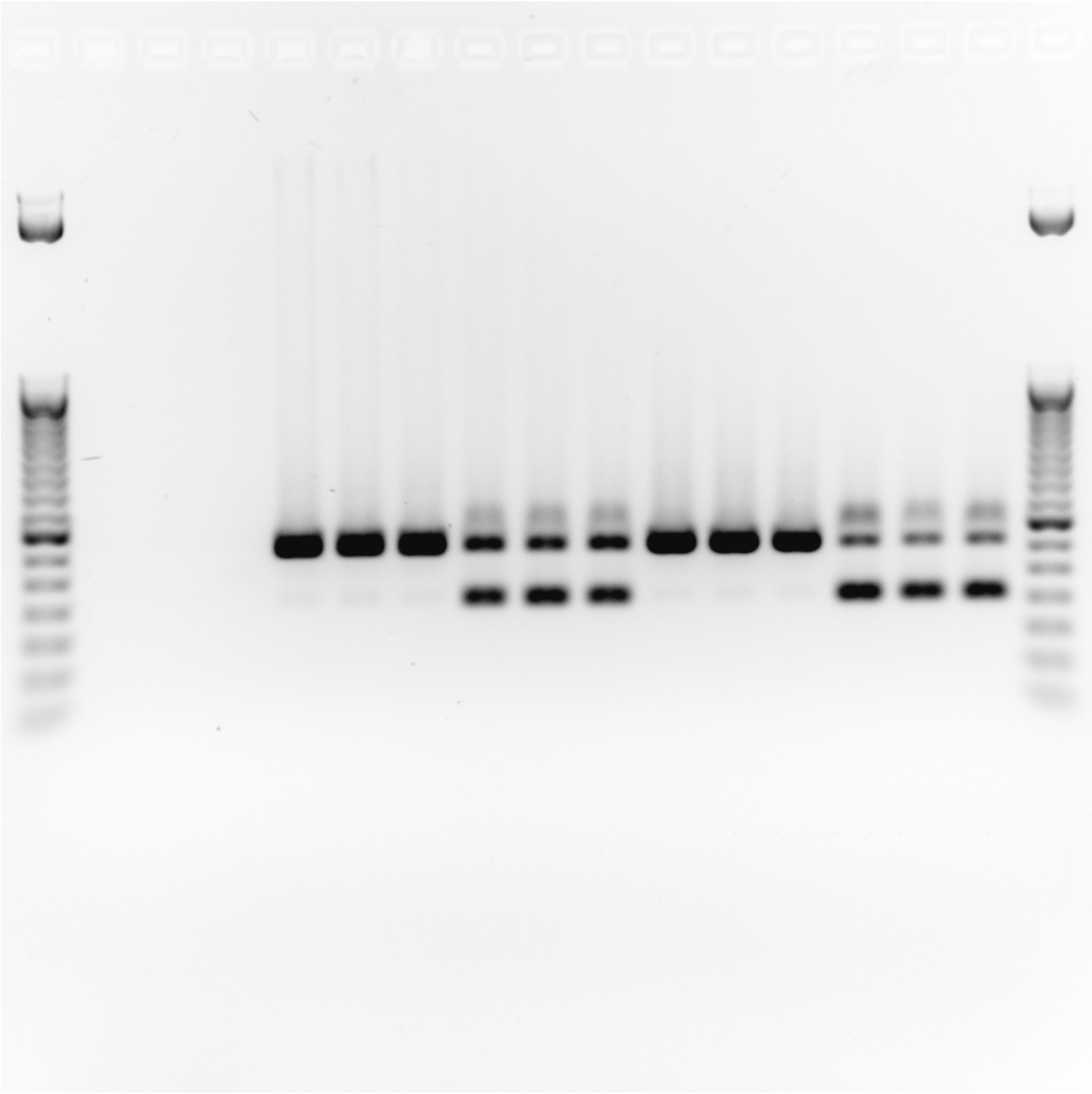

A.

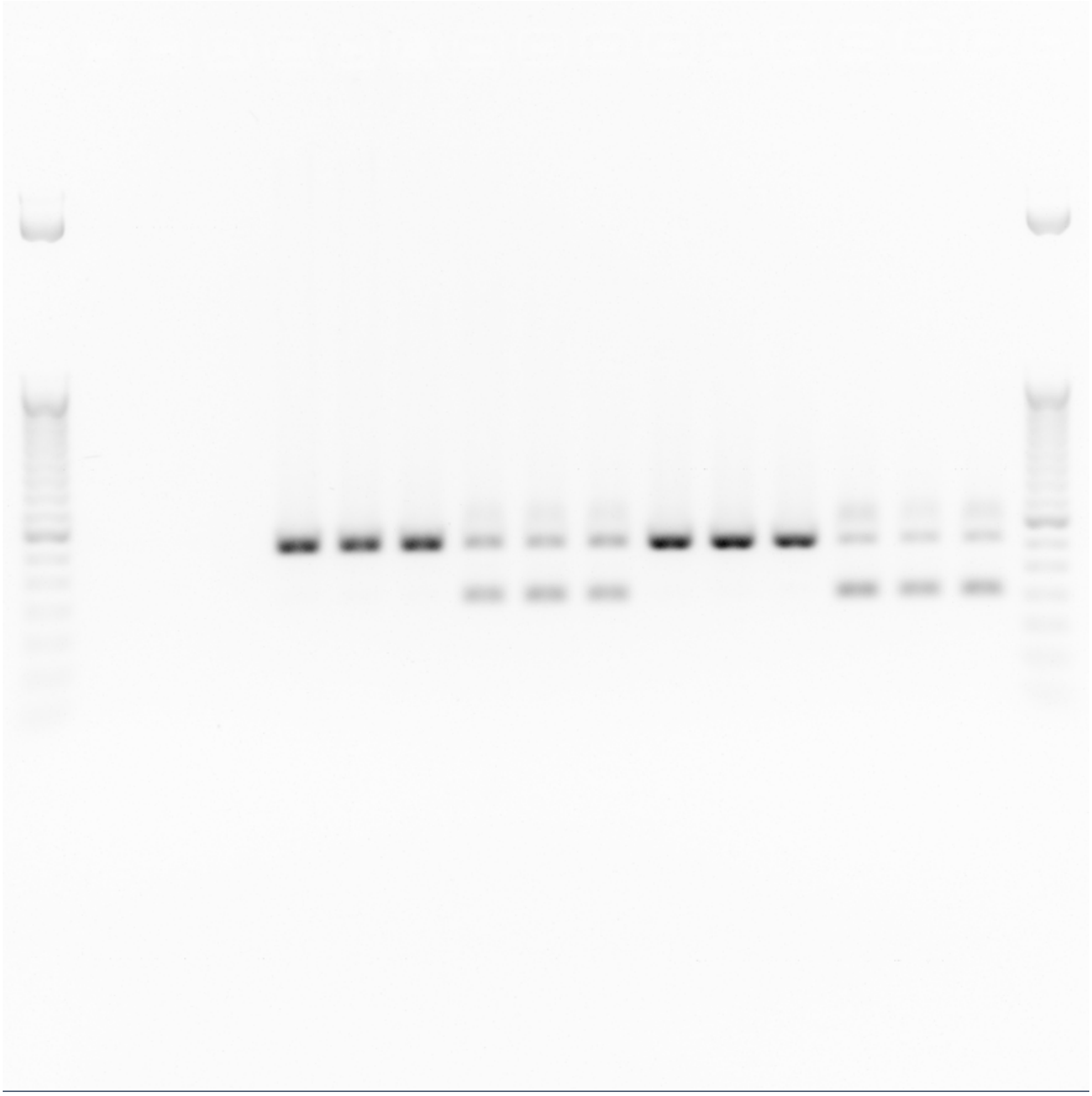

B.

C.

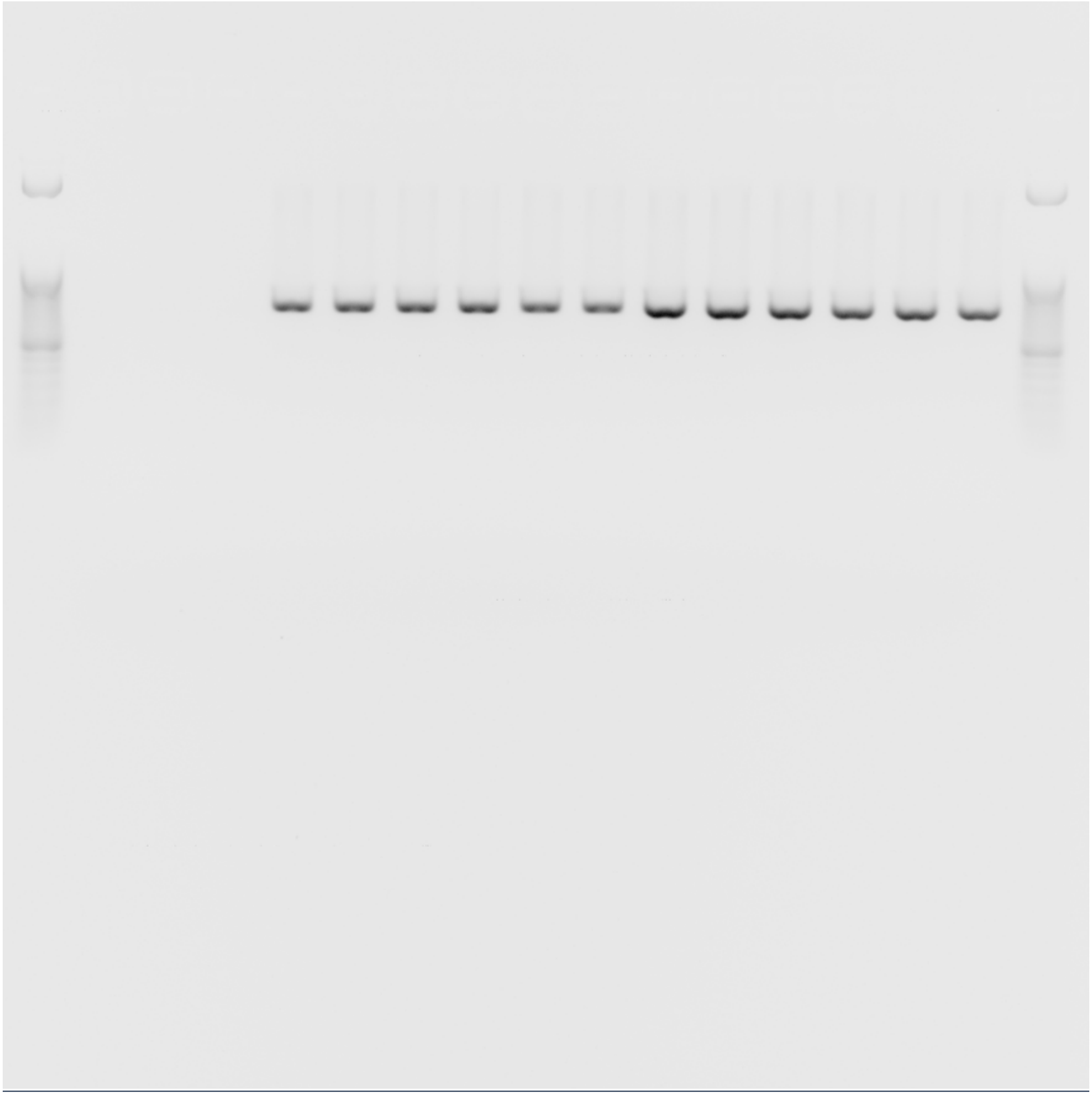

Supplementary Figure 7: Full gel images of those presented in S Fig 3. A) ACP1 B) mKate2

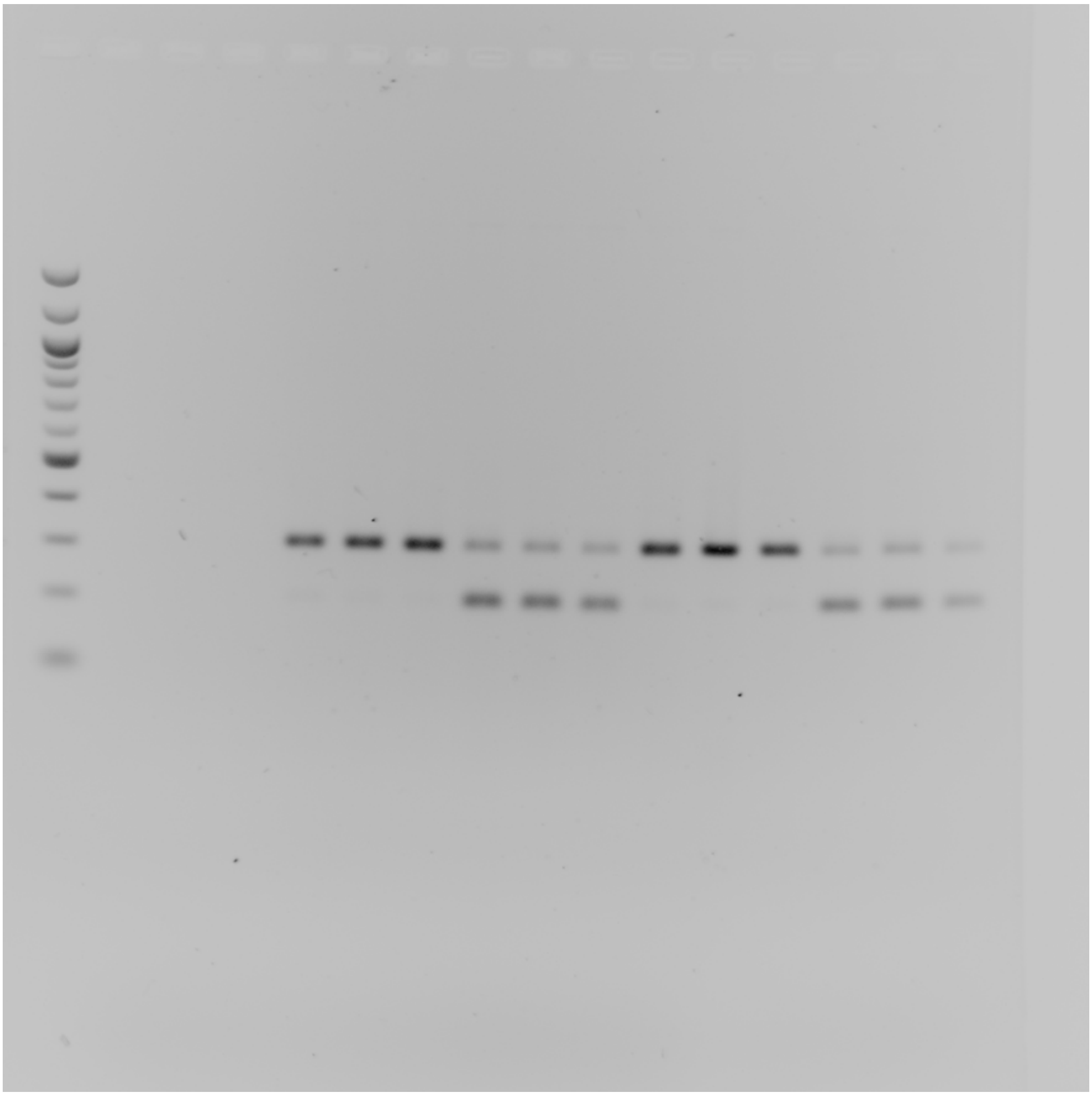

A.

B.

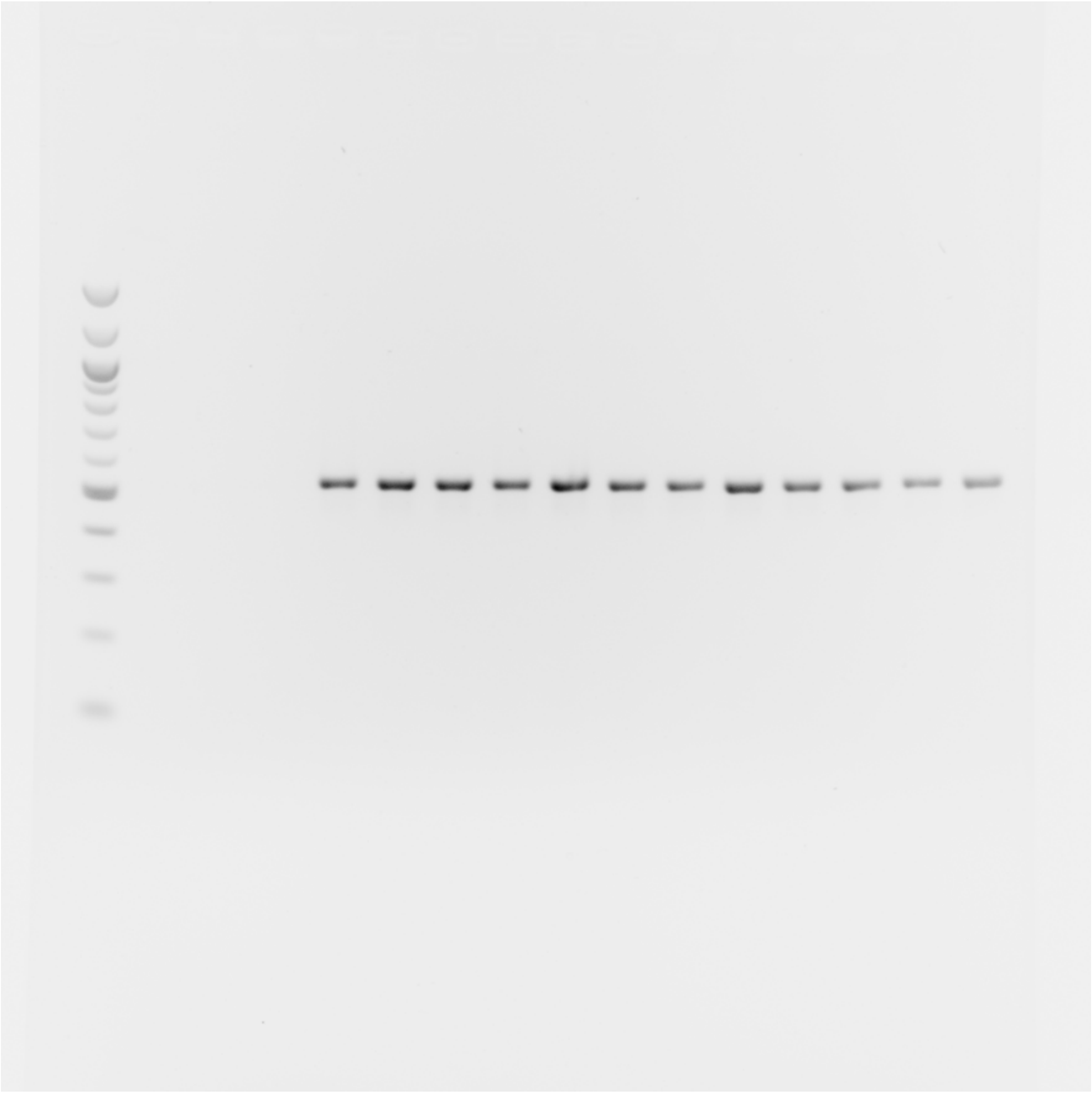

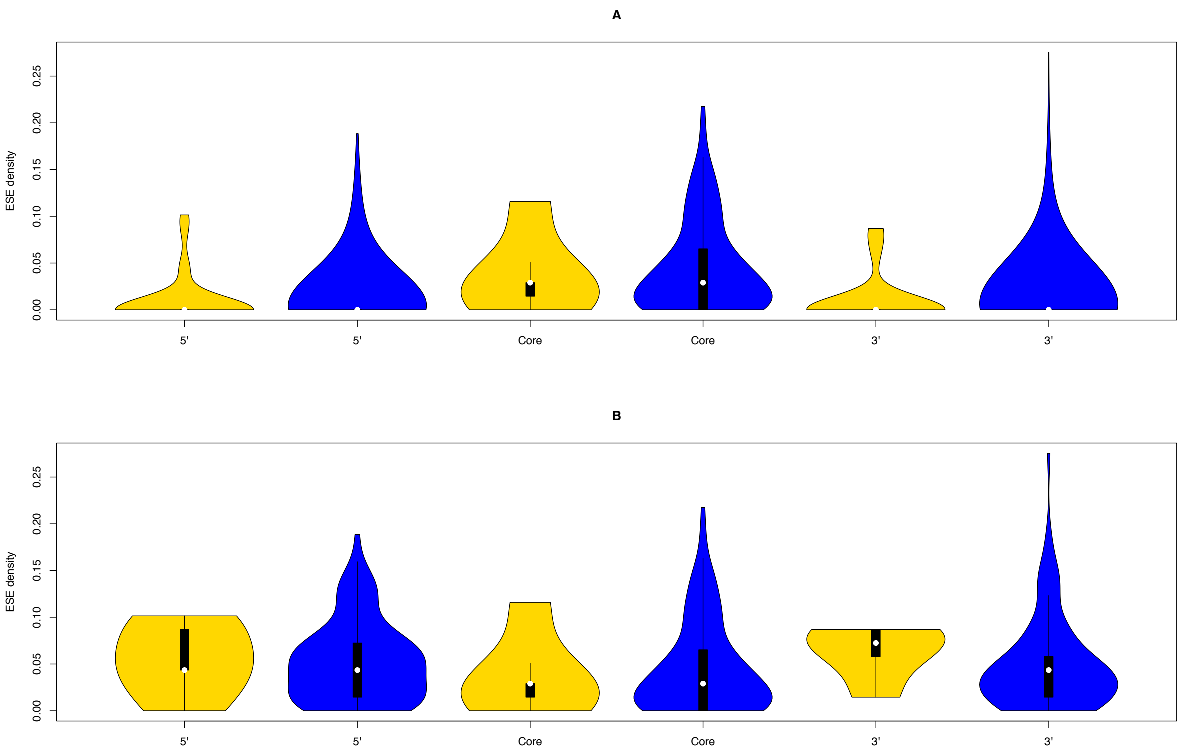

Supplementary Figure 8. ESE density in three exonic compartments for the NAS candidates (yellow) and non-NAs candidates (blue). A. all exons. There are 30 NAS exons and 511 non NAS exons. For each exon over 138 bp long the 5' and 3' ends were defined as the terminal 69bp. For those shorter than 138 bp the exon was cut in two and the two ends ascribed as either 5' or 3'. For those exons greater than or equal to 3 x 69 bp =207bp) the central 69 segment was defined as the core (n=5 for the NAS set, N=111 for the non NAS set). ESE density was defined as number of occurrences of all ESEs within the INT3 set per bp of sequence. Within each exonic compartment there are no differences in ESE density between the NAS and non NAS groups: Mann Whitney U test: 5' exon end: *P*=0.54; core *P*=0.84; 3' end *P*=0.93). Within each group there is heterogeneity between the exonic compartments (Kruskal Wallis test: NAS candidates, *P*=00056; non candidates $P<2 \times 10^{-16}$). **B. only exons >= 207 bp.** The above result compares ESE density across exons of all sizes, including those in which the core rate cannot be measured. Restricting analysis to the 116 long exons (5 NAS, 111 not NAS), results in the classical result that core has a lower density than the flanks. In a paired test, mean density of an exons' 5' and 3' flanks - core density of the same exon, the flanks have a higher density (mean difference = 0.014, N=116) the effect being on the edge of significance (paired t test P=0.07). Within each exonic compartment there are no differences in ESE density between the NAS and non NAS groups: Mann Whitney U test: 5' exon end: *P*=0.22; core *P*=0.22; 3' end *P*=0.27). This test is however underpowered given the small sample size of the NAS group.

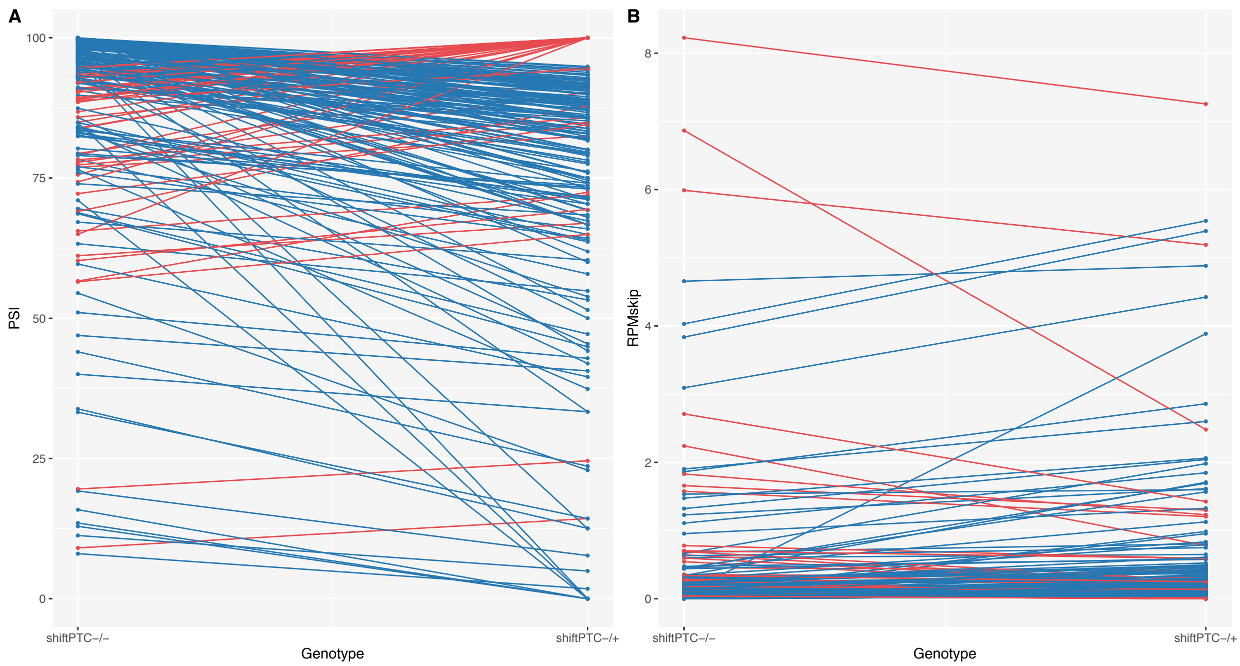

Supplementary Figure 9: Changes in PSI for large effect variants (>5%) for PTCs found off frame by one nucleotide.

(A) 171/218 of the large effect off-frame cases demonstrate a decrease in PSI for the PTC containing isoform, a significant number (P = 2.2×10^{-16}, one-tailed exact binomial test). (B) 183/218 of the shifted variants show an increase in RPMskip associated with the shiftPTC, again a significant number (P = 2.2×10^{-16}, one-tailed exact binomial test). 100/218 large effect shifted PTCs have both PSI and RPMskip in the direction consistent with NAS, arguing against an exclusively reading frame dependant mechanism of skipping.

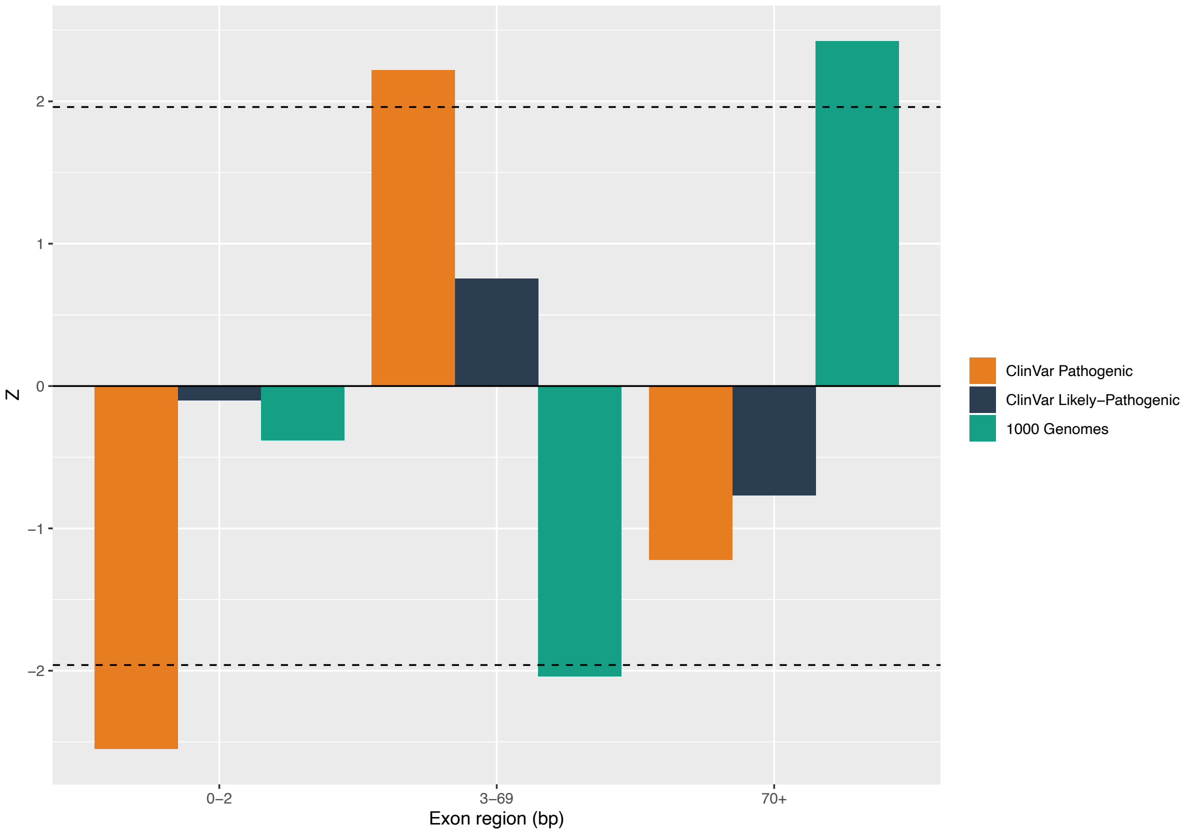

Supplementary Figure 10: Z scores for the number of nonsense mutations located in each exon region for the 1000 Genomes and ClinVar datasets when compared with randomly sampled nucleotide-matched simulants.

Z scores for the number of nonsense mutations located in each of the exonic regions when compared with 10,000 reference-allele nucleotide matched simulants for the pathogenic and likely-pathogenic ClinVar and the 1000 Genomes variants. The dotted line represents $Z = \pm 1.96$ where $P \approx 0.05$. Only the pathogenic variants are significantly enriched in the exon flank regions consistent with splice disruption being a consistent source of disease. The significantly negative 1000 Genomes Z (-2.039) is also consistent with these mutations segregating in a healthy population.
